# Flexible active-site loops fine-tune substrate specificity of hyperthermophilic metallo-oxidases

**DOI:** 10.1007/s00775-023-02040-y

**Published:** 2024-01-16

**Authors:** Vânia Brissos, Patrícia T. Borges, Ferran Sancho, Maria Fátima Lucas, Carlos Frazão, Felipe Conzuelo, Lígia O. Martins

**Affiliations:** 1 Instituto de Tecnologia Química e Biológica António Xavier, Universidade Nova de Lisboa, Av da República, 2780-157 Oeiras, Portugal; 2 Zymvol Biomodeling, C/ Pau Claris, 94, 3B, 08010 Barcelona, Spain

**Keywords:** Multicopper oxidases, Laccases, Enzyme engineering, Directed evolution, Enzyme specificity

## Abstract

Hyperthermophilic (‘superheat-loving’) archaea found in high-temperature environments such as *Pyrobaculum aerophilum* contain multicopper oxidases (MCOs) with remarkable efficiency for oxidizing cuprous and ferrous ions. In this work, directed evolution was used to expand the substrate specificity of *P. aerophilum* McoP for organic substrates. Six rounds of error-prone PCR and DNA shuffling followed by high-throughput screening lead to the identification of a hit variant with a 220-fold increased efficiency (*k*_cat_/K_m_) than the wild-type for 2,2'-azino-bis(3-ethylbenzothiazoline-6-sulphonic acid) (ABTS) without compromising its intrinsic activity for metal ions. The analysis of the X-ray crystal structure reveals four proximal mutations close to the T1Cu active site. One of these mutations is within the 23-residues loop that occludes this site, a distinctive feature of prokaryotic MCOs. The increased flexibility of this loop results in an enlarged tunnel and one additional pocket that facilitates bulky substrate-enzyme interactions. These findings underscore the synergy between mutations that modulate the dynamics of the active-site loop enabling enhanced catalytic function. This study highlights the potential of targeting loops close to the T1Cu for engineering improvements suitable for biotechnological applications.

**Graphical Abstract:**

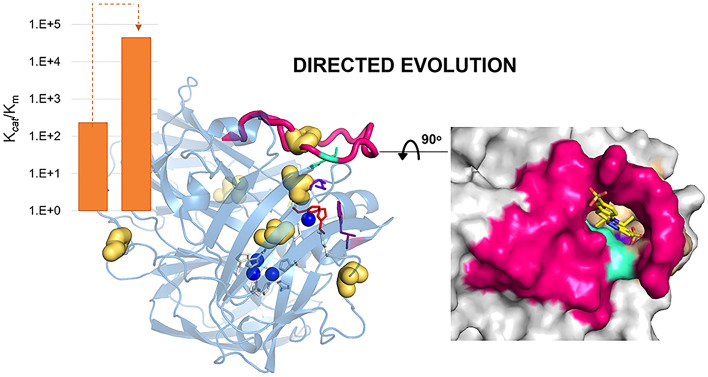

**Supplementary Information:**

The online version contains supplementary material available at 10.1007/s00775-023-02040-y.

## Introduction

**Scheme 1:**
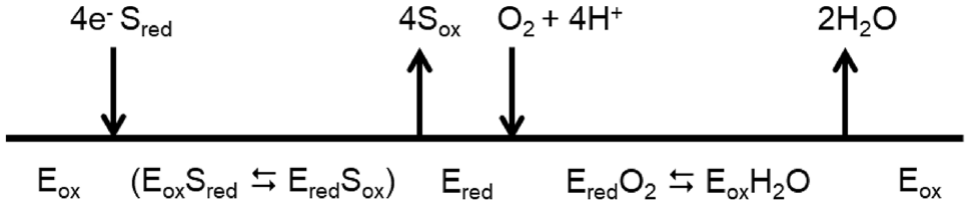
The general reaction of substrates oxidation and oxygen reduction by Mcos

Multicopper oxidases (MCOs) are proteins composed of three Greek key β-barrel cupredoxin domains (domains 1, 2, and 3) that come together to form three spectroscopically different Cu sites: type 1 (T1), type 2 (T2) and the binuclear type 3 (T3) [1–3]. MCOs couple the one-electron oxidation of substrates at the T1CU with the four-electron reduction of molecular oxygen to water at the T2/T3 trinuclear copper (TNC). Two histidine nitrogen atoms and a cysteine sulfur coordinate the T1CU [4]. The T3 coppers are coordinated by three histidines and a bridging ligand, such as a hydroxyl moiety; two histidine residues and a water (or hydroxyl) molecule coordinate the T2 copper site, strategically positioned close to the T3 binuclear Cu site. The catalytic mechanism of MCOs involves (i) the reduction of the T1CU site by the oxidized substrates, (ii) electron transfer (ET) from the T1CU site to the TNC via a conserved His-Cys-His pathway, and (iii) O_2_ reduction at the TNC [5] (Scheme 1). The substrates of MCOs vary from organic compounds to metal ions such as Fe(II), Mn(II), and Cu(I) and the family is generally divided into two classes, metalloxidases and laccases. Laccases, the most studied MCOs, oxidize aromatic substrates such as polyphenols, methoxy-substituted phenols, and amines and have found biotechnological applications in several industrial fields [6, 7]. Metallo-oxidases, such as yeast Fet3P and human ceruloplasmin (hCp), exhibit a robust activity towards low-valent transient metals such as Cu(I), Fe(II), and Mn(II) and are important in cellular metal homeostasis systems [8, 9]. Notably, laccases and metallo-oxidases have nearly identical Cu active sites but exhibit substantial diversity in substrate interactions and catalytic rates. Therefore, the investigation of structure–function relationships that explain differences in reactivity is crucial to provide both fundamental and technological insight.

**Fig. 1:**
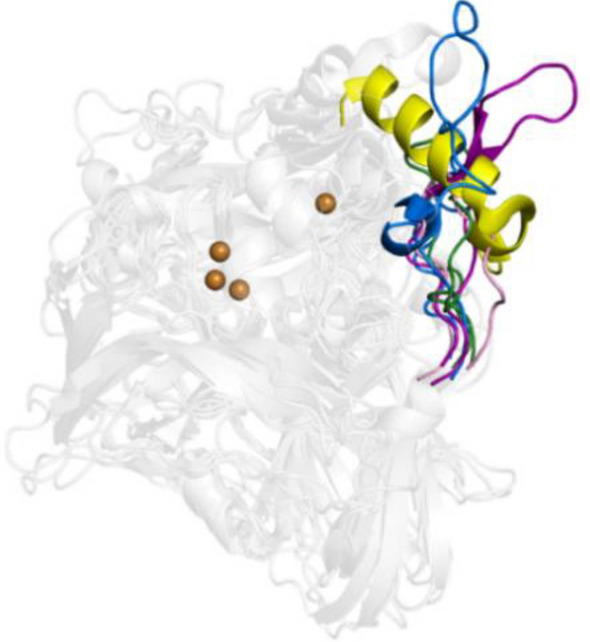
Superimposition of cartoons highlighting the major secondary structure elements occluding the access to the T1Cu center in prokaryotic MCOs, *Escherichia coli* CueO (yellow, PDB 1KV7), *Campylobacter jejuni* McoC (light pink, PDB 3ZX1), *Pyrobaculum aerophilum* McoP (green, PDB 3AW5), *Thermus thermophilus* Tth (purple, 2XU9), and *Aquifex aeolicus* McoA (blue, PDB 6SYY). Copper ions are represented as dark orange spheres

In laccases, the T1CU site occupies a depression on the surface of the third domain so that one of its histidine ligands can act as a primary electron acceptor to a large variety of substrates [10, 11]. In metallo-oxidases, the T1CU is not easily accessible to reducing substrates [12–15]. A striking feature of prokaryotic metallo-oxidases is the presence of methionine-rich segments partially occluding the T1Cu (Fig. 1) [13–17]. The mechanistic features of Met-rich segments in MCOs are largely unknown; in *Escherichia coli* CueO, an enzyme involved in cellular metal detoxification, the Met-rich loop was claimed to provide additional ligands to Cu(II)-binding sites [18–20]. In *Aquifex aeolicus* metallo-oxidase McoA [21], the Met-loop is a malleable Ω-loop lid-domain that interacts with the surrounding in an open-to-closed equilibria that modulates the catalytic activity [15]. Furthermore, optimizing the enzymatic function for aromatics was associated with alterations of the Met-loop conformational landscape [22, 23], pointing to a critical role of protein dynamics in guiding the evolution of new enzyme functions [24–26].

In this study, we engineered the hyperthermophilic metallo-oxidase *Pyrobaculum aerophilum* McoP [27] to enhance its activity for aromatics, resembling laccase activity, using directed evolution (DE). DE is a powerful tool for tailoring biocatalysts with improved features and has significantly contributed to our understanding of protein function and evolution [28–33]. Our results demonstrate that the enhanced catalytic activity of McoP for aromatics is linked to modifications in the flexibility of the 23-residue loop near the T1CU site and a reconfiguration of the substrate-binding cavity. These findings reinforce the significance of loops in prokaryotic MCOs that are proximal to the T1CU site, as they play a crucial role in shaping the active site and regulating turnover rates.

## Material and methods

### Chemicals and materials

All chemicals were of analytical grade and used as received. Ferrocenemethanol (> 95%) was from TCI Europe. Sulfuric acid (1 N) was from Carlo Erba. Citric acid monohydrate was from Merck. Di-sodium hydrogen phosphate dihydrate was from VWR Chemicals. 2-(N-morpholino) ethane sulfonic acid (MES) was from Sigma-Aldrich. The O_2_ concentration in the solution was measured using a dissolved oxygen measurement station (Hanna Instruments). All solutions were prepared with ultrapure deionized water from a Milli-Q purification system (*ρ* = 18 MΩ cm).

### Bacterial strains, plasmids, and culture media

*E. coli* strain DH5α (Novagen) was used to amplify plasmid constructs. *E. coli* Tuner (DE3, Novagen) strain in which the cueO gene that codes for the multicopper oxidase [34] is inactivated (*E. coli* Tuner ΔcueO::kan) was used to express the *mcop* gene cloned in pET-21a (+) plasmid (Novagen) (pVB1) or its evolved variants. In the Tuner strain, the *mcoP* gene is controlled by the T7 promoter, and its expression is induced by isopropyl β-D-1-thiogalactopyranoside (IPTG). Luria–Bertani medium (LB) and Terrific Broth medium (TB) were used to grow *E. coli* strains, supplemented with appropriate antibiotics.

### Construction of *mcoP* wild-type without signal peptide sequence

The signal peptide present in the N-terminus of McoP was removed in the wild-type enzyme using the plasmid pATF-2 [21] as a template, and the primers mcoP-R29M 5'- and mcoP-1637R (Table S1). The R29M mutation was introduced to create a new starting codon. PCR was carried out in a 50 µL reaction volume containing a 3 ng DNA template, one µM of primers, 200 µM of dNTPs, NZYProof polymerase buffer, and 2.5 U of NZYProof polymerase (NZYTech). After an initial denaturation period of 5 min at 94 °C, the following steps were repeated for 20 cycles in a thermal cycler (MyCyclerTM thermocycler, Biorad): 1 min at 94 °C, 1 min at 55 °C, 2 min at 72 °C followed by a final 10 min period at 72 °C. The amplified products were purified using GFX PCR DNA and Gel Band Purification Kit (GE Healthcare). The final PCR products were digested with *Nde*I/*EcoR*I (Thermo Scientific), cloned into pET-21a (+) (Novagen), and the construct pVB1 was introduced into electrocompetent *E. coli* DH5α cells. The absence of inadvertent mutations in other inset regions was confirmed by DNA sequencing using T7 terminator universal primers.

### Random mutagenesis by error-prone PCR and mutant library construction

Primers mcoP-R29M and mcoP-1637R were used for amplification. Ep-PCR was performed in a 50 μl final volume containing 3 ng of DNA template (pVB1), one μM of each primer, 0.2 mM dNTPs, 1.5 mM MgCl_2_, *Taq* polymerase buffer, and 2.5 U *Taq* Polymerase (Thermo Scientific). The effect of MnCl_2_ has been tested at 0.01–0.2 mM concentrations. After an initial denaturation period of 5 min at 94 °C, the following steps were repeated for 25 cycles in a thermal cycle (Mycycler thermocycler, Biorad): 1 min at 94 °C, 1 min at 55 °C and 2 min at 72 °C followed by a final 10 min period at 72 °C. The amplified products were purified using the GFX PCR DNA and the Gel Band Purification Kit (GE Healthcare). The amplified products were digested with *Nde*l*/EcoR*I (Thermo Scientific) and cloned into plasmid pET-21a (+) (Novagen). These pET-21a (+) plasmids expressing *mcoP* evolved variants were introduced into electrocompetent *E. coli* Tuner ΔcueO::kan cells.

### Recombination by DNA shuffling and variant library construction

DNA shuffling was performed with the genes coding for the *mcoP* wild-type without the signal peptide and 1B5 variant as previously described [35]. The final PCR products were digested with *Nde*I/*EcoR*I (Thermo Scientific), cloned into pET-21a (+) (Novagen), and introduced into electrocompetent *E. coli* Tuner ΔcueO::kan cells.

### “Activity-on-plate” high-throughput screening

*E. coli* Tuner Δ*cueO*::*kan* cells harboring expression plasmids were grown overnight on solid LB or TB medium supplemented with ampicillin (100 µg L^−1^), kanamycin (10 µg L^−1^), and 0.05 mM IPTG. Colonies were replica-plated onto chromatography paper (Whatman) as described previously [23]. In brief, the colonies on the filter papers were carefully lysed with lysozyme (0.5 µg mL^−1^) in 20 mM Tris–HCl buffer, pH 7.6. The filter papers were incubated for 2 h at 37 °C. Then the filter papers were soaked in a solution containing 2.5 mM CuCl_2_ to allow proteins to bind copper, and 1–20 mM 2,2ʹ-azino-bis(3-ethylbenzothiazoline-6-sulphonic acid) (ABTS) in 100 mM acetate buffer, pH 4.5 and incubated overnight at 43 °C. Their green or purple color identified the variants showing increased enzymatic activities.

### High-throughput activity screening in 96-well plates

Colonies were picked from the original transformation plates and transferred to 96-well plates containing 200 μL of LB medium supplemented with ampicillin (100 ug L^−1^) and kanamycin (10 μg L^−1^). Cell cultivation, disruption and activity screenings in crude extracts for ABTS were performed as previously described [23]. Variants showing higher activity than the parental strain were rescreened to eliminate false positives. Mutations were verified by DNA sequencing analysis using T7 terminator universal primers. Each generation’s variant with the highest activity was selected as the next generation's parent.

### Construction of *mcoP* variants using site-directed mutagenesis

Single amino acid substitutions in the *mcoP* gene were created using the Quick-change mutagenesis protocol (Stratagene). Plasmid containing the *mcoP* gene without signal peptide (pVB1) was used as a template with appropriate primers (Table S1). PCRs were carried out in 50 µL reaction volumes containing 3 ng of DNA template, 2 μM of primers, 200 µM of dNTPs, NZYProof polymerase buffer, and 1.25 U of NZYProof polymerase (NZYTech). After an initial denaturation period of 5 min at 94 °C, the following steps were repeated for 25 cycles in a thermal cycler (MyCycler™ thermocycler, Biorad): 1 min at 94 °C, 2 min at 57–72 °C, 10 min at 72 °C followed by a final 10 min period at 72 °C. The amplified product was purified using GFX PCR DNA and a Gel Band Purification Kit (GE Healthcare). The final PCR product was first digested with *Dpn*I (Thermo Scientific) to eliminate the wild-type template and then used to transform electrocompetent *E. coli* KRX cells. The presence of the desired mutation in the resulting plasmid and the absence of additional inadvertent mutations in other regions of the inset were confirmed by DNA sequencing.

### Production and purification of McoP wild-type and variants

Electroporation introduced gene coding for the wild-type and variant constructs into the *E. coli* Tuner ΔcueO::kan host strain. Culturing recombinant *E. coli* strains was performed as previously described [23]. Cell cultures were collected by centrifugation (18,000 × *g*, 15 min at 4 °C). Cell pellets were suspended in 20 mM Tris–HCl buffer, pH 7.6, containing 5 mM MgCl_2_ and 1 U mL^−1^ of DNAse I. Cells were disrupted by French Press (Thermo EFC) and lysates centrifuged at 40,000 × *g* for 2 h at 4 °C. Cell crude extracts were incubated at 80 ºC for 20 min, and denatured proteins removed by centrifugation (12,000 × *g* for 10 min). The resultant supernatants were loaded onto an ion exchange Q-Sepharose column equilibrated with 20 mM Tris–HCl, pH 7.6. McoP was eluted without absorption. The active fractions were pooled, concentrated, and loaded onto a Superdex 200 HR 10/30 column equilibrated with 20 mM Tris–HCl buffer, pH 7.6, containing 0.2 M NaCl. After washing the column with the same buffer, the active fractions were pooled, and enzyme concentration was estimated using the molar absorption coefficient of McoP (*ε*_280_ = 57 750 M^−1^·cm^−1^), calculated from the protein sequence using the ExPASy Bioinformatics Resource Portal (http://web.expasy.org) or the Bradford assay using bovine serum albumin as standard. In vitro, copper incorporation was performed as previously described [22, 36].

### Kinetic analysis and stability assays

The activity dependence on pH was measured by monitoring the oxidation of ABTS at 420 nm (*ε* = 36,000 M^−1^ cm^−1^) and of DMP at 468 nm (*ε* = 49,600 M^−1^ cm^−1^) at 40 °C using Britton-Robinson buffer (100 mM phosphoric acid, 100 mM boric acid and 100 mM acetic acid mixed with NaOH to the desired pH in the range 3–10). Apparent steady-state kinetic parameters were measured by adding the enzyme to a mixture containing ABTS (0.02 − 30 mM) and 100 mM acetate buffer, DMP (0.02 − 5 mM) and 100 mM phosphate buffer at optimal pH, and ferrous ammonium sulfate (0.04–2 mM) and MES buffer pH 5 at 315 nm (*ε* = 2200 M^−1^ cm^−1^). Cuprous oxidase activity was estimated at 40 °C by measuring oxygen consumption (Oxygraph; Hansatech) [21]. Kinetic data were fitted directly using the Michaelis − Menten equation using Origin® software. All enzymatic assays were performed at least in triplicate. Thermodynamic stability was performed using differential scanning calorimetry (DSC) measurements as previously described [23, 27].

### Electrode modification

Gold disk electrodes (2 mm diameter, CH Instruments) were used; before modification, electrode surfaces were polished using diamond polishing pastes (LECO) of decreasing particle size (0.5 µm and 0.1 µm) and alumina polishing paste (0.05 µM, BASi). After cleaning, rinsing, and drying, the electrodes were electrochemically cleaned by cyclic voltammetry in 0.5 M H_2_SO_4_, sweeping between 0.0 V and 0.6 V vs. Ag/AgCl/3.5 M KCl at 100 mV^−1^. The cleaned electrode surface was modified by drop casting with 1 μL of protein (3.0 mg mL^−1^, unless otherwise specified). The modified electrodes were dried at room temperature for 30 min and rinsed with buffer before use.

### Electrochemical measurements

All experiments were performed in a three-electrode configuration, using the modified electrode as the working electrode, a Pt wire as a counter electrode, and a commercial (TjAida) Ag/AgCl/3.5 M KCl reference electrode (*E* = 0.2046 V vs. SHE). Electrochemical characterization was performed by cyclic voltammetry using an SP-300 potentiostat (BioLogic) within a predefined potential range at a scan rate of 10 mV s^−1^. Unless otherwise noted, the third cycle of a consecutive series of potential sweeps is presented. The electrodes were measured in air-equilibrated buffer solutions (O_2_ concentration measured to be 0.26 mM) or after bubbling the solution with N_2_ for at least 20 min (O_2_ concentration < 5 µM). All measurements were performed at room temperature.

### Crystallization

Crystallization trials of the 3F3 variant (in 20 mM Tris–HCl pH 7.6 supplemented with 200 mM NaCl) were performed with Structure Screen I and II (Molecular Dimensions) at 20 ºC. Crystallization drops were set using a Mosquito crystallization robot (SPT Labtech) with 96-well sitting drop iQ plates. The Structure Screen I and II screens produced 3F3 crystals within two days at 20 ºC in a 2.0 M Ammonium sulfate crystallization solution, 0.1 M Sodium HEPES pH 7.5, and 2% (v/v) PEG 400. The crystallization hits were further optimized at micro-liter scale by the hanging drop vapor-diffusion method, using XRL 24-well crystallization plates (Molecular Dimensions) and 500 μL of precipitant in the reservoir different precipitant concentrations, protein: precipitant ratios and different temperatures were tested. The best 3F3 crystals appeared after one week at 20 ºC with 2.0 M Ammonium sulfate, 0.1 M Sodium HEPES pH 7.5, and 7% (v/v) PEG 400. The needle-like crystals showed a light blue color. The crystals were transferred to the reservoir solution supplemented with 20% (v/v) glycerol before being flash-cooled in liquid nitrogen.

### Data collection and processing

Datasets for 3F3 crystals were collected at the ALBA synchrotron (Barcelona, Spain) on beamline BL13-XALOC [37]. Diffraction images of the 3F3 variant were recorded with a PILATUS 6 M detector using 0.97926 Å wavelength radiation, 576.34 mm crystal-to-detector distance, and 0.15º oscillations widths, a total of 135º rotation. Diffraction data were integrated and scaled with XDS [38]. 3F3 variant crystal datasets were processed in space group P4_1_2_1_2. Data collection details and processing statistics are in Table S2.

### Structure determination and refinement

3F3 variant unit cell contained two molecules in the asymmetric unit, corresponding to a *V*_M_ of 2.59 Å^3^ Da^−1^ and a solvent content of ~ 53% [39, 40]. The 3F3 crystal structure was solved by molecular replacement with MoRDa [41] using the coordinates of the previously published crystal structure of *P. aerophylum* McoP wild-type (PDB 3AW5 [16] as the search model (*Q* score = 0.885). The structure was refined with PHENIX.REFINE [42–44]. Random intensities (1.5%) were excluded from monitoring the refinement strategy. Though refinement comprised standard stereochemistry libraries [45], the inter-atomic distances involving copper centers and their ligands were refined without target restraints. The TLSMD server (http://skuld.bmsc.washington.edu/~tlsmd) [46] defined structural regions of translation, libration, and screw (TLS) refinement. Refinement cycles included atomic coordinates and individual isotropic *a.d.p.s*, TLS refinement, and automatic solvent waters modeling, with hydrogen bonding distances within 2.45–3.40 Å. Refinement cycles were complemented with model comparison against 2 m|Fo|-D|Fc| and m|Fo|-D|Fc| electron density maps in COOT [47]. MOLPROBITY [48] was used to analyze the stereochemical quality of the system, and the accessible surface area (ASA) was calculated using AREAIMOL [49–51]. The structure figures were prepared using PyMOL [52, 53]. Cavities were determined using Dogsitescorer [54] and tunnels using MOLE 2.0 [55]. Refinement statistics are presented in Table S2. The experimental structure factors and atomic coordinates were available in the Protein Data Bank (www.rcsb.org) with PDB code 8P4G.

### Docking simulations

Before docking, residues 296–302, which are part of the loop 288–310 and are not visible in the electron density maps of the 3F3 variant, were modeled using the Google Colab platform and AlphaFold2_advanced option https://colab.research.google.com/github/sokrypton/ColabFold/blob/main/beta/AlphaFold2_advanced.ipynb#scrollTo=Riekgf0KQv_3, accessed on 22 March 2023 [56], refined using the Amber-relax option to enhance the accuracy of the side chains geometry. The predicted local-distance difference test (pLDDT) confidence values (higher = better) are indicated in the B-factor column. The 296–302 residues were selected from the AlphaFold2 model and inserted into the 3F3 crystal structure. Local energy minimization was applied in this region with Yasara [57] using NOVA as a force field [58]. Moreover, and preceding docking, the protonation states of the titrable residues were determined by PROPKA [59]. For the ABTS, it was implemented a global charge of -2, and all dockings simulations were performed with Yasara using the Autodock Vina [60]. First, to find the most significant ABTS binding sites, a cell of 2 Å was applied around the whole proteins at pH 4, the optimal pH of the enzyme. Docking simulations were carried out for rigid side chain receptors using YAMBER as a force field [61]. Then, the docking solutions were analyzed through a clustering process based on RMSD calculations (2 Å). A more restrictive docking was performed where a cell of 10 Å was centered on the T1Cu, the most populated substrate-binding region of these enzymes. To identify the enzyme: ABTS complex systems, the docking solutions were scored and filtered according to the binding energy vs*.* the distances between the ABTS and the different residues in the T1Cu site that could be involved in the substrate binding. Moreover, the Pathways VMD plugin was used to calculate the ET rates to determine the possible residues involved from the ABTS (atoms N1 and N2) to the T1Cu [62]. The docking solutions were scored and filtered by the distance between ABTS and H292, H294, W355, or H465 to ensure an optimal first residue of the electron pathway. Next, results were analyzed according to the binding energy vs*.* the electron transfer rate involved in the ET from ABTS to T1Cu. Figures showing the docking results were created with PyMOL [52, 53].

### Other methods

UV–visible absorption spectra were obtained as previously described [27]. The copper content was determined through the trichloroacetic acid/bicinchoninic acid method [63].

## Results and discussion

### Directed evolution towards increased activity for organic compounds

**Fig. 2:**
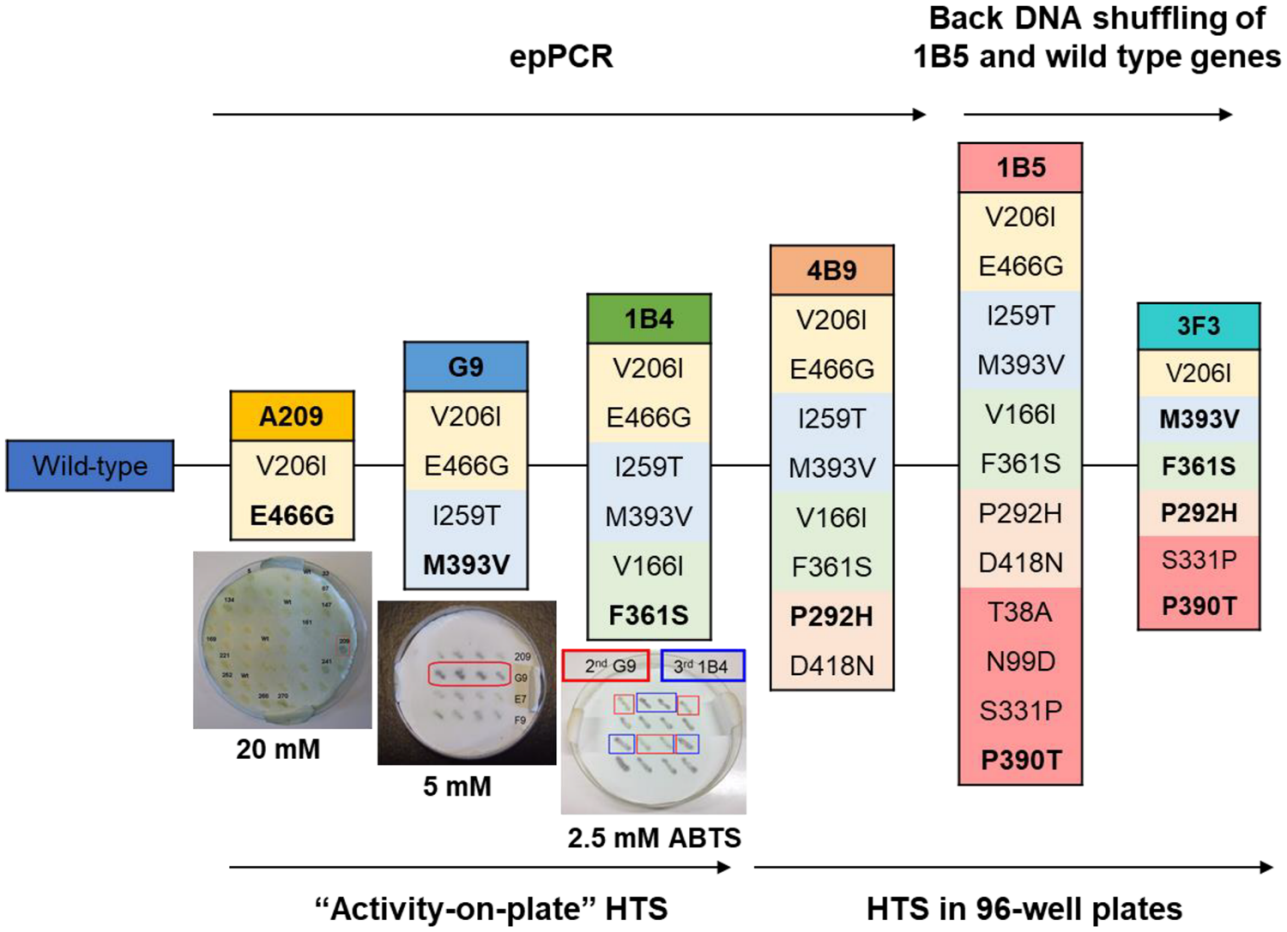
Lineage of McoP variants after six rounds of directed evolution. In the 1st round, ~ 20,000 clones were screened using “activity-on-plate” with 20 mM ABTS as substrate. A209 was chosen as a parent for the second round of evolution, and ~ 7000 clones were screened using 5 mM ABTS as substrate. Variant G9 was selected for the third round, where ~ 12,000 variants were screened using 2.5 mM ABTS. Variant 1B4 was selected for the fourth round, where ~ 13,000 clones were screened using 2.5 mM ABTS as substrate, and 810 variants showing the highest activity were rescreened in liquid assays in 96-well plates. Variant 4B9 was chosen for the fifth round of evolution, where ~ 10,000 variants were screened using 2 mM of ABTS as substrate, and 436 variants were rescreened in 96-well plates. Variant 1B5 was selected, and after DNA-shuffling with wild-type gene, ~ 4000 variants were screened using 2 mM of ABTS as substrate, 378 variants were rescreened in 96-well plates, and variant 3F3 was selected. We have observed the introduction of at least one mutation close to the copper centers in each round of evolution: E446G, M393V, F361S, P292H, and P390T (in bold; see Fig. 3)

We have first deleted the TAT-dependent signal peptide (MITRRRFLQIGLGAGAMLAMGFTLQYILR) of McoP to improve the amounts of soluble protein [16]. The resulting enzyme shows full copper content (ratio Cu/protein of 3.8:1) and the typical MCOs’ spectroscopic properties (Table S3) [27]. Five rounds of DE using error-prone PCR (ep-PCR) generated approximately 65,000 clones (Fig. 2) that were screened for ABTS activity using high-throughput assays [23]. The hit variant 1B5 of the fifth generation contained 12 mutations; to reduce to the smallest subset of mutations, the last round of evolution was performed with DNA-shuffling of genes coding for wild-type and 1B5 [64]. A library of variants (~ 4200 clones) was screened for ABTS activity, and variant 3F3, with only six mutations, was selected for further studies (Table S4).

### Biochemical and kinetic analyses

The best hit of each generation was produced in *E. coli*, purified, and characterized (Table S3). The catalytic properties of purified variants were determined at the optimal pH (Fig. S1) using ABTS as substrate (Table 1). Variant 1B5 from the 5th generation increased about 70-fold higher specificity (*k*_cat_/K_m_) for ABTS compared to the wild-type (Table 1). The 3F3 variant selected after DNA shuffling showed a ~ tenfold higher *k*_cat_ than the 1B5 and a remarkable 2-order of magnitude higher *k*_cat_/K_m_ compared to wild-type. Notably, the turnover number for ABTS of 3F3 is close to the one measured for the model bacterial CotA-laccase (*k*_cat_ = 144 s^−1^) [65]; these results significantly surpass the outcomes of a similar engineering approach, where a McoP variant featuring the four mutations G225S, L284H, F290I, and T341A demonstrated only a ninefold increase in enzymatic activity compared to wild-type [66]. The introduced mutations did not have any discernible impact on thermal stability. All variants exhibited melting temperatures around 100 °C, consistent with the wild-type enzyme (Table 1 and Fig. S2). This suggests the absence of a trade-off between functionality and stability, a phenomenon often encountered during directed evolution.

**Table 1:** Apparent steady-state kinetic parameters for ABTS (100 mM sodium acetate at optimal pH) and melting temperatures (T_m_) by differential scanning calorimetry (DSC) of the purified wild-type and variants

Enzymes	pH_op_	*k*_cat_ (s^−1^)	*K*_m_ (mM)	*k*_cat_/*K*_m_ (s^−1^·M^−1^)	*T*_m_ (ºC)
Wild-type	3.5	1.60 ± 0.02	7.1 ± 0.5	(0.2 ± 0.02) × 10^3^	105 ± 2
A209	3.8	2.4 ± 0.4	6.4 ± 0.4	(0.4 ± 0.1) × 10^3^	105 ± 2
G9	4.4	3.2 ± 0.2	0.4 ± 0.1	(0.8 ± 0.2) × 10^4^	102 ± 2
1B4	4.4	3.3 ± 0.1	1.0 ± 0.1	(0.3 ± 0.3) × 10^4^	95 ± 2
4B9	4.8	6.4 ± 0.4	0.4 ± 0.1	(1.6 ± 0.4) × 10^4^	94 ± 3
1B5	4.8	19 ± 2	1.4 ± 0.2	(1.4 ± 0.3) × 10^4^	95 ± 2
3F3	4.0	113 ± 11	2.6 ± 0.1	(0.44 ± 0.05) × 10^5^	105 ± 1

Reactions were performed at 40 ºC. *T*_m_ values were calculated using the first derivative of enthalpy (*C*_p_)

The analysis of the single variants showed that the mutations introduced in the first three rounds of evolution, E466G, M393V, and F361S, lead only to a ~ twofold higher *k*_cat_ to ABTS when in the wild-type background relative to that of wild-type (Tables S5 and S6). In contrast, mutations P390T and P292H from the fourth and fifth rounds of evolution have a higher impact on enzyme fitness, resulting in a 7- and 16-fold higher activity than wild-type. This result is consistent with previous engineering studies where mutations introduced later in the evolution show the strongest functional impact [22]. The cumulative effect of all mutations in the wild-type background would led to only a modest theoretical ~ 20-fold increase in *k*_cat_/K_m_, suggesting non-additive interactions between mutations, a phenomenon known as epistasis, since a remarkable 220-fold increase was experimentally determined in the 3F3 variant. The wild-type enzyme displayed residual activity with the lignin-related phenolic substrate 2,6 dimethoxyphenol (DMP), and the evolution process resulted in a more than tenfold increase in *k*_cat_/K_m_ for the 3F3 variant, with values of 124 vs. 9.5 s^−1^ M^−1^ (Table S7).

Furthermore, we have determined the catalytic parameters of 1B5 and 3F3 variants for Cu(I) and Fe(II), which are the presumed physiological substrates of the metallo-oxidase McoP (Table 2 and Fig. S3); both hit variants exhibited approximately 5- to 15-fold higher catalytic efficiency towards Cu(I) and an impressive 4-orders of magnitude improvement with Fe(II) indicating that the introduced mutations have significantly enhanced the enzyme's overall catalytic fitness.

**Table 2:** Apparent steady-state kinetic parameters for Cu(I) (100 mM sodium acetate buffer, pH 3.5) and Fe(II) (100 mM MES buffer, pH 5) of the purified wild-type and hit variants

Enzymes	Cu(I)			Fe(II)		
	*k*_cat_ (s^−1^)	*K*_m_ (mM)	*k*_cat_/*K*_m_ (s^−1^·M^−1^)	*k*_cat_ (s^−1^)	*K*_m_ (mM)	*k*_cat_/*K*_m_ (s^−1^·M^−1^)
Wild-type	11 ± 2	0.11 ± 0.05	(1 ± 0.1) × 10^5^	0.013 ± 0.002	1.2 ± 0.4	11 ± 1
1B5	54 ± 9	0.10 ± 0.04	(5.4 ± 0.3) × 10^5^	–	–	(0.9 ± 0.1) × 10^5^
3F3	71 ± 11	0.05 ± 0.01	(14 ± 0.1) × 10^5^	–	–	(1.7 ± 0.2) × 10^5^

Reactions were performed at 40 ºC

### Structural analysis

**Fig. 3:**
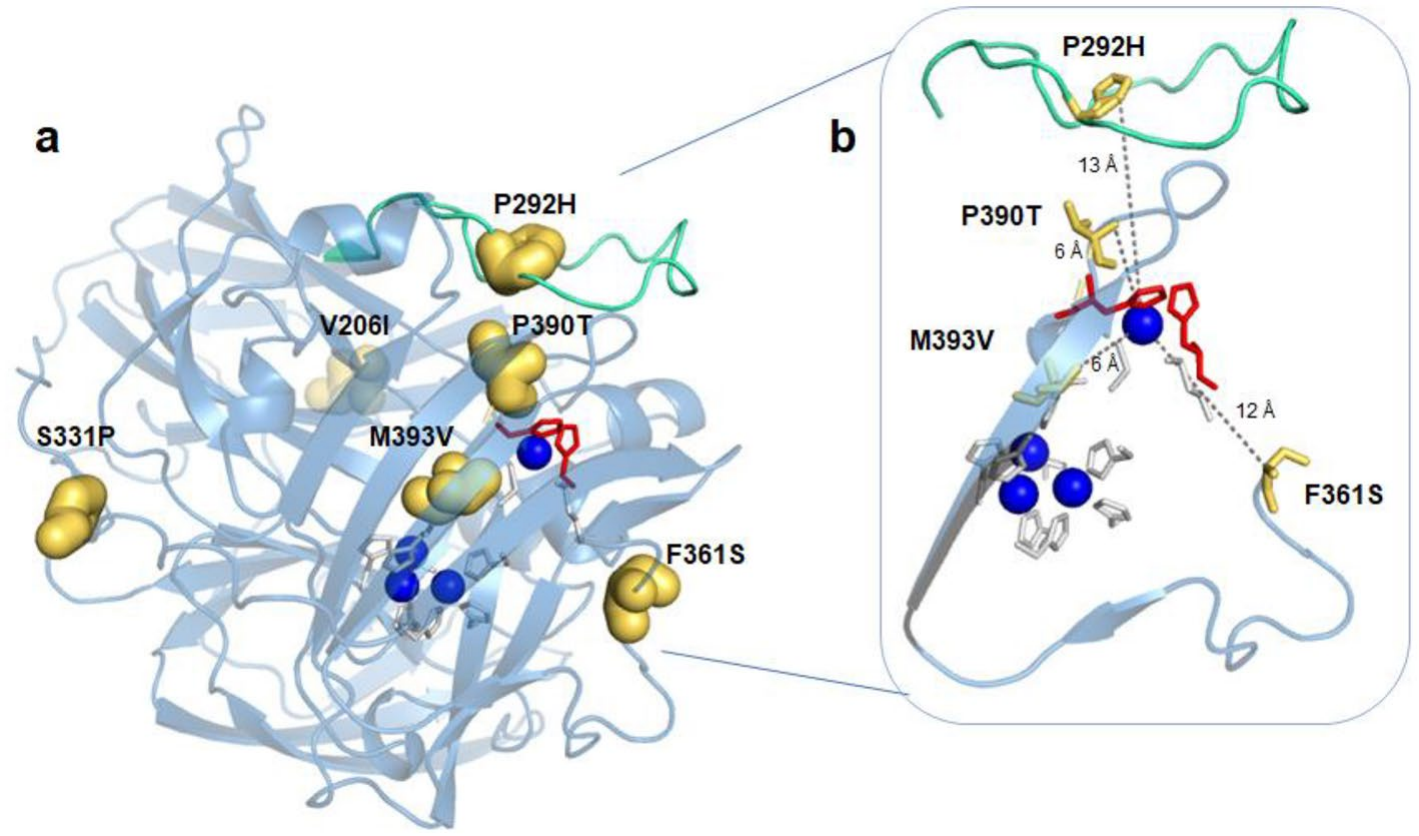
Mapping mutations in the 3F3 variant. **a** Transparent cartoon representation of the 3F3 crystal structure colored in blue. The mutations V206I, P292H, S331P, F361S, P390T, and M393V are thick golden sticks. **b** Zoomed view of the closest mutations to the T1Cu, displayed as thin golden sticks. The loop 288–310 is colored in green cyan. The copper atoms are represented in Klein blue spheres. The T1Cu ligands (H391 and H465) and TNC are in red and grey sticks, respectively. The mutations P390T and M393V, closer to T1Cu, show the lowest accessible surface areas (ASA), 8% and 1%, respectively, whereas the remaining mutations display ASA values between 29 and 39%

The crystal structure of the 3F3 evolved variant show two molecules in the asymmetric unit (Table S2 and Fig. S4) and a root-mean-square deviation (RMSD) of 0.47 Å between their Cα positions to the wild-type structure (PDB 3AW5); furthermore, the T1Cu and TNC ligands have a similar conformation to the wild-type (Fig. S4). Mutations V206I and S331P are distal (at > 20 Å) whereas the remaining are closer to the T1Cu (Fig. 3); P390T (adjacent to the T1Cu ligand H391 and M393V (adjacent to the T2Cu ligand H394) are at ~ 6 Å to the T1Cu, F361S at ~ 12 Å and P292H at ~ 13 Å. Given that substrate oxidation in MCOs takes place at the T1Cu site, it is highly probable that alterations in substrate specificity are primarily influenced by structural variations in proximity to this site.

In McoP, a 23-residue loop ^288^TPFDHMHLEMGHGMQEALPEGSE^310^ and a short α-helix (188–195) are occluding the T1Cu. The overall average B-factor value of 3F3 (49 Å^2^) is higher than the wild-type enzyme (32 Å^2^) (Fig. S5), and the loop 288–310 in the 3F3 is the region that shows higher flexibility, with B-factor values ranging from 43–69 Å^2^ (vs*.* 22–58 Å^2^). Part of this loop (segment ^296^EMGHGMQ^302^) is not even visible in the electron density maps and was modeled with AlphaFold2 applying a local energy minimization using Yasara (Fig S6). A structural superposition revealed that the loop 288–310 of McoP is replaced in the metallo-oxidase *A. aeolicus* McoA (PDB 6SYY) by a long methionine-rich unstructured loop, 327–355, not visible in the electron density maps [15].

Prolines are known for their exceptional conformational rigidity. The observed increase in flexibility of the loop in the 3F3 variant may be attributed to structural rearrangements resulting from mutations in the loop, such as P292H, as well as mutations close to the T1CU site, like P390T (Fig. 3). Furthermore, their replacement introduced two polar residues in a hydrophobic neighboring. However, it is important to note that crystal packing could also influence the increased flexibility observed. For instance, the longer distance of M353 in chain B to segment 296–302 in chain A may induce additional destabilization in this region. Recently, it was reported that mutation F290I in McoP, located in the loop T288-E310, improved the turnover rate for ABTS by around sixfold (28 s^−1^ at 50 °C) [66]; the X-ray crystal structure analysis (PDB 6K3D) suggested that this mutation increased the flexibility of the loop, improving the ET between the enzyme and the substrate.

McoP was recently utilized to fabricate biocathodes employing various electrodes and immobilization strategies [66–69]. In this study, wild-type McoP and the 3F3 variant were adsorbed onto gold electrodes (AuEs). Surprisingly, we observed that the electrochemical response of the wild-type enzyme (17 ± 1 µA cm-2) was significantly higher than that of the 3F3 evolved variant (current responses of 3.8 ± 0.6 µA cm-2) (Fig. S7). To explore whether the kinetic limitation observed in the 3F3 variant stems from suboptimal interactions with the electrode surface, we analysed in the presence of ferrocenemethanol (FcMeOH), a freely diffusible redox mediator (Fig. S8); however, the electrocatalytic response showed only a partial enhancement. Previous studies with *E. coli* CueO have suggested the importance of methionines located close to the T1 Cu for immobilizing the enzyme onto gold-surface electrodes [70, 71]. In the case of McoP, most exposed methionines are situated near the T1Cu (**Fig. S9 and Table S8**). Notably, three methionines (M293, M297, and M301) are part of the loop that shows increased flexibility in 3F3, while the other four methionines (M145, M190, M192, M353), are less exposed in the variant as compared to wild-type (7–27% vs. 28–56%). The reduced exposure of methionines in the 3F3 variant may have resulted in an increased distance between the electrode and the T1Cu, leading to less efficient ET and a significantly lower current response compared to the wild-type (Fig. S7).

**Fig. 4:**
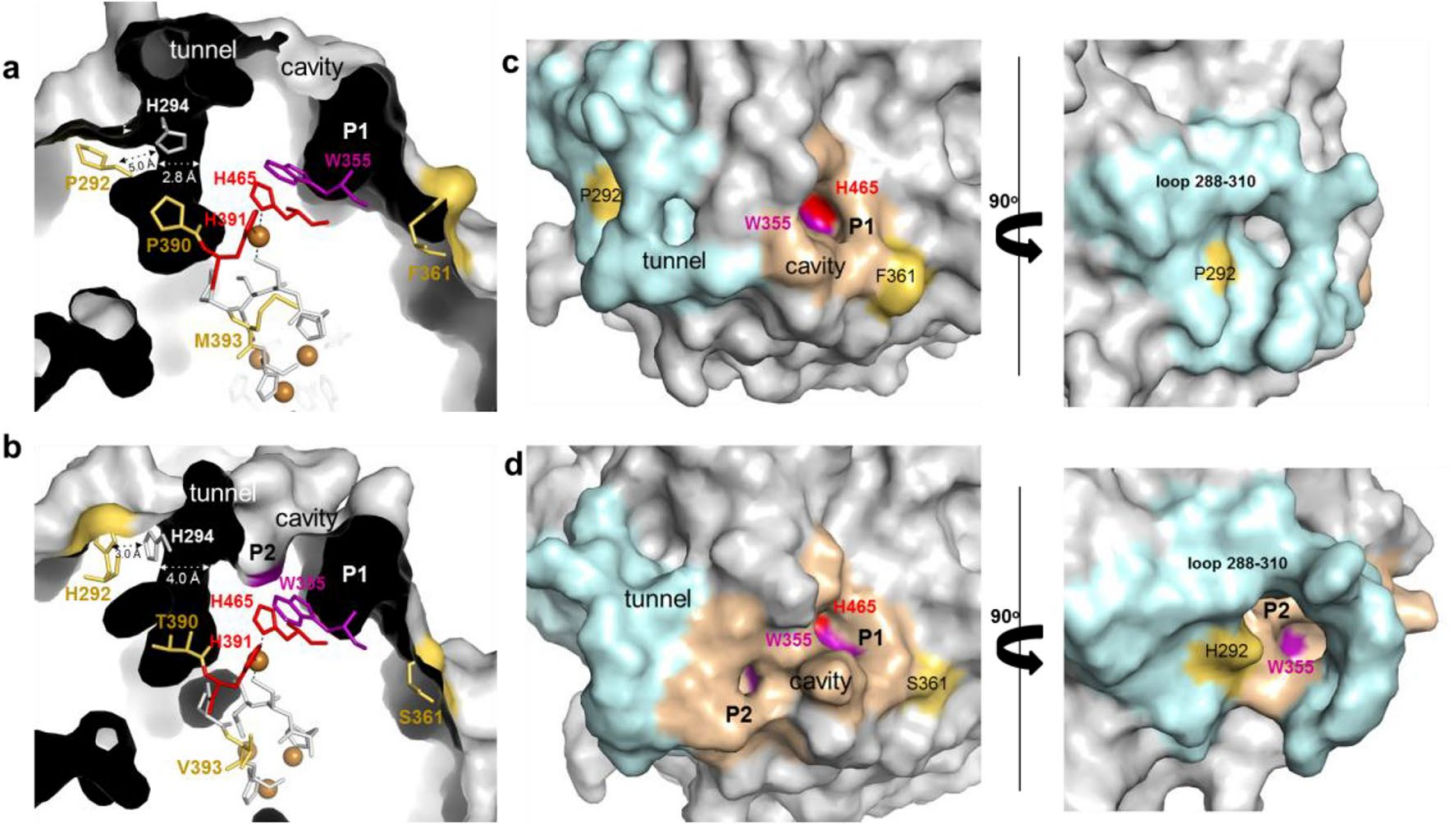
Molecular access to the T1Cu center. Cutaway view of the solvent-accessible surface in wild-type (PDB 3AW5) (**a**) and variant 3F3 (**b**) showing the tunnel and cavity (with pockets P1 and P2). The T1Cu histidine ligands (H391 and H465) are red, while the other TNC ligands are grey. The W355 residue is shown as purple sticks. The mutated residues P292H, F361S, P390T, and M393V are labeled as yellow. The diameter of tunnels, in wild-type and 3F3 variant, and the distance H294-H292 are shown as light grey dashed lines. The solvent-accessible surface of McoP (**c)** and variant 3F3 (**d**) shows the loop 288–310 (blue cyan) surrounding the tunnel. The cavity (beige) shows solvent-exposed T1Cu ligands H465 (red) and W355 (purple). The pocket P1 is labeled in wild-type (**c**), and both P1 (exposing H465 and W355) and P2 (exposing W355) are visible in 3F3 (**d**). Surface mutations P292H and F361S are shown in yellow. A rotation of 90 degrees in the y-axis (right panel in (**d**)) allows a more precise view of the P2 main entrance in the 3F3 variant, while in wild-type, it is occluded (right panel in (**c**))

The analysis of the solvent access to the T1Cu in the crystal structures allows the definition of a cavity and a tunnel that putatively facilitates ET to the T1Cu (Fig. 4, Table S9 and S10). The increased flexibility of the loop 288–310 in 3F3 caused structural alterations at the entrance of the tunnel: it is more polar than in the wild-type (Fig. S10) and has a larger diameter access entry (4.0 Å vs. 2.8 Å) due to the shorter distance between H294 and H292 (3 Å vs. 5 Å) (Fig. 4a, b, and Table S9). In the wild-type enzyme, access to T1Cu is facilitated through a pocket (P1) that forms part of the cavity and exposes H465, which serves as a ligand to T1Cu (Fig. 4a, c). In 3F3, W355, which is located at a distance of 3.4 Å from H465, exhibits significantly greater exposure to the solvent than in the wild-type. Its ASA increases from 7 to 22 Å^2^ in 3F3. This increased exposure is attributed to the higher flexibility of the loop 288–310 (Fig. S11), leading to the emergence of a new pocket (P2). This new pocket may facilitate ET transfer from substrates to T1 and contribute to the improved enzymatic activity observed in the variant (Fig. 4b, d). Interestingly, during the evolution of the metallo-oxidase McoA to enhance activity for organic substrates, a new pocket for ABTS emerged [22].

### Docking simulations

**Fig. 5:**
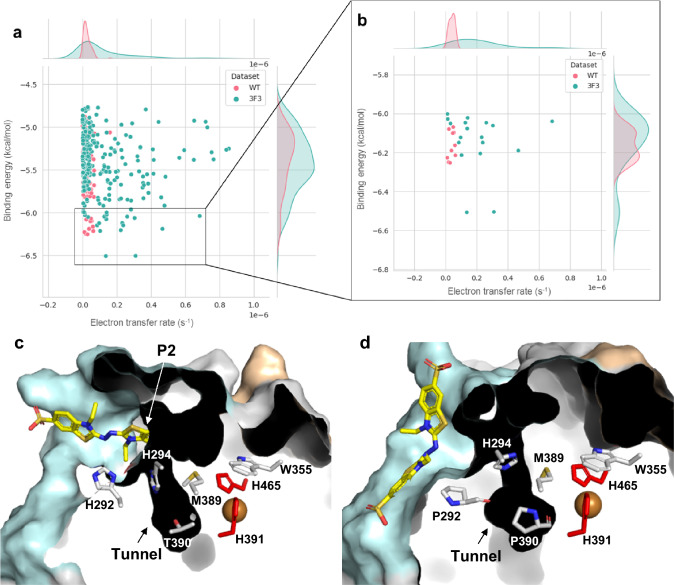
Docking ABTS to wild-type and 3F3 variant. Binding energy (kcal/mol) vs. electron transfer rate is shown for wild-type and 3F3 variant (**a**). The critical substrate positions for the wild-type are colored pink, while those for the 3F3 variant are colored blue. Only docking positions closer than 3.0 Å to suitable solvent-exposed residues near T1Cu (H292, H294, W355, or H465) were considered. A zoomed view of the most efficient ABTS binding positions for ET is shown (**b**). Cutaway view of the solvent-accessible surface of representative enzymes with lower binding energy and higher ET rates: variant 3F3 (**c**) and wild-type (PDB 3AW5) (**d**), showing the possible residues involved in ET. The T1Cu histidine ligands (H391 and H465) are colored in red, while the carbon atoms of the other residues are shown in grey. The oxygen and nitrogen atoms are colored in red and blue, respectively. The copper atom is shown as an orange sphere

Docking ABTS in 3F3 shows binding modes closer to the T1Cu with slightly lowest binding energies compared to the wild-type (Fig. 5a, b and Fig. S12). The analysis of the binding energy vs. distances of ABTS-G469 (a residue defining P1) (Fig. S12b) and of ABTS-H294 (a residue defining P2) (Fig. S12c) reveals that in the wild-type, ABTS predominantly populates close to P1, whereas in the 3F3 variant, it tends to be closer to P2. Qualitative ET rate calculations have revealed in 3F3 variant that the most efficient ET is obtained when residues H294, M389, W355, and H465, which define part of P2, are involved in the ET, with a rate that can reach approximately 1 × 10^−6^ (Fig. 5b, c). In the wild-type, the most efficient pathway involves residues P292, P390, and H391, although with ET rates tenfold lower (~ 0.1 × 10^−6^; Fig. 5b, d). These lower ET values are consistent with the wild-type residual enzymatic activity for ABTS. Overall, the data suggests that in 3F3, P2 may serve as an ABTS binding site, facilitating the transfer of electrons to the more exposed T1Cu, resulting in a remarkable 200-fold increase in catalytic efficiency. As mentioned, both enzymes exhibit limited competent ABTS docking positions near P1 (Fig. 5a, b). This limitation may be attributed to the carboxylate residues, such as E464 and D467, in this region, which could impair the productive binding of the negatively charged ABTS molecule. Consequently, we hypothesize that P1 is not involved in ABTS binding but may play a role in ET from metal ions Cu(I) and Fe(II). This hypothesis aligns with what was observed in proteins like the yeast ferroxidase Fet3p, where carboxylate residues (E185, D409, and D283) coordinate the uptake and subsequent transfer of electrons from substrates to T1CU [8]. However, this possibility was not investigated here.

## Conclusion

The rate-limiting step in MCOs is the oxidation of the substrate at the T1CU site. The first and second-sphere residues surrounding this site are crucial in controlling intermolecular ET from the substrate to T1 and intramolecular ET from T1 to the trinuclear copper center (TNC). The significant increase in specificity for ABTS observed in the evolved 3F3 variant can be attributed to the introduction of mutations that resulted in conformational reconfigurations of a tunnel and cavity, facilitating access to the T1Cu site. Notably, this also led to an additional pocket within the substrate-binding cavity, where in silico*,* productive ABTS binding occurred more frequently. These structural alterations were associated with rearrangements in the flexible loop typically located near T1Cu centers in metallo-oxidases from the MCOs family. These findings align with previous observations in *A. aeolicus* McoA, where the Met-loop interacts with the surroundings to regulate access to the T1Cu site and facilitate interactions with substrates [15, 21]. They underscore the critical role of active-site loops close to the T1Cu site in shaping the structure–function relationships that enable multi-functionality within MCOs. Moreover, these results highlight the efficacy of engineering loops surrounding the T1Cu site as a potent strategy for customizing hyperthermophilic metallo-oxidases to accommodate relevant organic substrates. This is particularly valuable considering the robustness of these enzymes, which enables them to operate under extreme conditions —an attribute of great importance in industrial processes. Such advancements support the development of more environmentally sustainable and efficient processes.

### Supplementary Information

Below is the link to the electronic supplementary material.

Supplementary information: Table with bacterial strains, plasmids, and primers (Table S1); X-ray data collection, processing, and refinement statistics (Table S2). Copper content and molar coefficients at 600 nm for wild-type McoP and variants (Table S3); Sequence analysis, activity and stability of the most active variants obtained after DNA-shuffling (Table S4); Copper content, molar coefficients at 600 nm and apparent kinetic parameters of wild-type and variants (Tables S5-S7); Solvent accessible surface area (ASA) of the methionines residues (Table S8); Dimensions and residues delimiting the tunnels and cavities (Table S9-10); Figures showing the pH profile of McoP and variants (Figure S1); Melting temperatures (Tm) of McoP and variants by differential scanning calorimetry (DSC) and temperature profile of McoP and 3F3 (Figure S2); Michaelis-Menten plots for metal ions oxidation (Figure S3); Overall structure of 3F3 and wild-type (Figure S4); B-factors representation (Figure S5); ASA of 3F3 with highlight in the flexible loop ((Figure S6); Voltammetric responses (Figures S7-S8); ASA of 3F3 and wild-type showing solvent-exposed methionines Met (Figure S9); Tunnel properties (Figure S10); Displacement of loop 288-310 in 3F3 (Figure S11); ABTS docking simulations (Figure S12)

## Data Availability

Data will be made available upon request.
