# Supplementary material for: Flexible active-site loops fine-tune substrate specificity of hyperthermophilic metallo-oxidases
Source: J Biol Inorg Chem. 2024 Jan 16;29(3):339–51. doi: 10.1007/s00775-023-02040-y (PMC11111587; doi:10.1007/s00775-023-02040-y)
Supplement: Supplementary file 1 — Supplementary information: Table with bacterial strains, plasmids, and primers (Table S1); X-ray data collection, processing, and refinement statistics (Table S2). Copper content and molar coefficients at 600 nm for wild-type McoP and variants (Table S3); Sequence analysis, activity and stability of the most active variants obtained after DNA-shuffling (Table S4); Copper content, molar coefficients at 600 nm and apparent kinetic parameters of wild-type and variants (Tables S5-S7); Solvent accessible surface area (ASA) of the methionines residues (Table S8); Dimensions and residues delimiting the tunnels and cavities (Table S9-10); Figures showing the pH profile of McoP and variants (Figure S1); Melting temperatures (Tm) of McoP and variants by differential scanning calorimetry (DSC) and temperature profile of McoP and 3F3 (Figure S2); Michaelis-Menten plots for metal ions oxidation (Figure S3); Overall structure of 3F3 and wild-type (Figure S4); B-factors representation (Figure S5); ASA of 3F3 with highlight in the flexible loop ((Figure S6); Voltammetric responses (Figures S7-S8); ASA of 3F3 and wild-type showing solvent-exposed methionines Met (Figure S9); Tunnel properties (Figure S10); Displacement of loop 288-310 in 3F3 (Figure S11); ABTS docking simulations (Figure S12) [file 775_2023_2040_MOESM1_ESM.docx]

**SUPPORTING INFORMATION**

**Flexible Active-Site Loops Fine-Tune Substrate Specificity of Hyperthermophilic Metallo-Oxidases**

Vânia Brissos^1^, Patrícia T. Borges^1^, Ferran Sancho^2^, Maria Fátima Lucas^2^, Carlos Frazão^1^, Felipe Conzuelo^1^, and Lígia O. Martins^1^

^1^*Instituto de Tecnologia Química e Biológica António Xavier, Universidade Nova de Lisboa, Av da República, 2780-157 Oeiras, Portugal.*

^2^*Zymvol Biomodeling, C/ Pau Claris, 94, 3B, 08010 Barcelona, Spain*

Correspondence: Lígia O. Martins, [lmartins@itqb.unl.pt](mailto:lmartins@itqb.unl.pt)

**Table S1.** Bacterial strains, plasmids, and primers, for the construction of McoP without the putative signal peptide and variants of McoP used in this study. F indicates forward primers, and R indicates reverse primers.

| **Strains, Plasmids, or Primers** | **Genotype, property, or sequence** | **Reference or source** |
| --- | --- | --- |
| ***E. coli* Strains** |  |  |
| DH5α | F^–^ φ80*lac*ZΔM15 Δ(*lac*ZYA a*rg*F)U169 *rec*A1 *end*A1 *hsd*R17(r_K_^–^, m_K_^+^) *pho*A *sup*E44 λ^–^ *thi*-1 *gyr*A96 *rel*A1 | Novagen |
| Tuner (DE3) | F^–^ *ompT hsdS*_B_ (r_B_^–^ m_B_^–^) *gal dcm lacY1*(DE3) | Novagen |
| TunerΔCueO (DE3) | TunerΔCueO::kan (*cueO* mutant obtained by kan^r^ gene replacement) | (Brissos et al. 2015) |
| **Plasmids** |  |  |
| pET21a(+) | Cloning vector with a T7 promoter with an N-terminal T7tag and C-terminal 6xHis tag; amp^r^ | Novagen |
| pATF2 | pET21a(+) with *mcoP* inserted into *Nde*I and *Eco*RI sites | (Fernandes et al. 2010) |
| pVB1 | pET21a(+) with *mcoP* without the signal peptide inserted into *Nde*I and *Eco*RI sites. The R29M mutation was introduced to create a new starting codon. | This work |
| pE466G | pET21a(+) with variant E466G inserted into *Nde*I and *Eco*RI sites. | This work |
| pM393V | pET21a(+) with variant M393V inserted into *Nde*I and *Eco*RI sites. | This work |
| pF361S | pET21a(+) with variant F361S inserted into *Nde*I and *Eco*RI sites. | This work |
| pP292H | pET21a(+) with variant P292H inserted into *Nde*I and *Eco*RI sites. | This work |
| pP390T | pET21a(+) with variant P390T inserted into *Nde*I and *Eco*RI sites. | This work |
| **Primers** |  |  |
| mcoP-R29M | 5'-ACATATGACTGGTGAAGTCAAGAGGCCTG-3' | This work |
| mcoP-1637R | 5'-CGCCGAGCGAATTCTTTAACTGC-3' | (Fernandes et al. 2010) |
| mcoP E446G F | 5’- CACAATTTAGAACATGGCGACGGGGGCATGATG -3' | This work |
| mcoP E466G R | 5’- CATCATGCCCCCGTCGCCATGTTCTAAATTGTG -3' | This work |
| mcoP M393V F | 5’- CGTCTATGCCCCACCCTGTGCACTTACACGGCTTTCC -3' | This work |
| mcoP M393V R | 5’- GGAAAGCCGTGTAAGTGCACAGGGTGGGGCATAGACG -3' | This work |
| mcoP F361S F | 5’- GACGATAAACGGCATGAGCTGGAATGCCTCAAACCC -3' | This work |
| mcoP F361S R | 5’- GGGTTTGAGGCATTCCAGCTCATGCCGTTTATCGTC -3' | This work |
| mcoP P292H F | 5’- GAATACGCCTTTTGATCATATGCATTTAGAAATGGGCC -3' | This work |
| mcoP P292H R | 5’- GGCCCATTTCTAAATGCATATGATCAAAAGGCGTATTC -3' | This work |
| mcoP P390T F | 5’- CGACAAGGCGTCTATGACCCACCCTATGCACTTACAC -3' | This work |
| mcoP P390T R | 5’- GTGTAAGTGCATAGGGTGGGTCATAGACGCCTTGTCG -3' | This work |
| mcoP F290I F | 5’- CTTGAAGAATACGCCTATTGATCCCATGCATTTAG -3' | This work |
| mcoP F290I R | 5’- CTAAATGCATGGGATCAATAGGCGTATTCTTCAAG -3' | This work |

**Table S2.** X-ray data collection, processing, and refinement statistics of the 3F3 variant. Values in parentheses belong to the highest resolution shell.

| **Data Collection** |  |
| --- | --- |
| Beamline | BL13-XALOC |
| Wavelength (Å) | 0.97926 |
| Space group | *P4_1_2_1_2* |
| Unit cell parameters (Å) | *a* = b= 128.4, *c* = 125.0 |
| Resolution (Å) | 73.45-2.59 (2.69-2.59) |
| Number of observations | 180537 (23148) |
| Unique reflections | 32726 (5178) |
| Completeness (%) | 99.1 (99.1) |
| Multiplicity | 5.5 (4.5) |
| Mosaicity (°) | 0.07 |
| CC_1/2_ (%)^a^ | 98.6 (32.2) |
| R_sym_ (%)^b^ | 12.9 (99.8) |
| R_meas_ (%)^c^ | 26.4 (200.5) |
| R_pim_ (%)^d^ | 9.0 (41.6) |
| <I/σ(I)> | 7.48 (0.76) |
| Wilson B-factor (Å^2^) | 48.9 |
| V_M_ (Å^3^ Da^-1^) | 2.59 |
| Estimated solvent content (%) | 52.57 |
| **Refinement** |  |
| R_work_ (%)^e^ | 21.3 |
| R_free_ (%)^e^ | 26.0 |
| r.m.s.d. for bond lengths (Å) | 0.002 |
| r.m.s.d. for bond angles (°) | 0.577 |
| Structure <*a.d.p.>* (Å^2^) | 49.1, 49.8 |
| Number of residues | 433, 434 |
| Number of solvent waters | 142 |
| Ramachandran plot |  |
| Residues in favored regions (%) | 96.9 |
| Residues in allowed regions (%) | 3.0 |
| Residues in disallowed regions (%) | 0.1 |
| PDB code | 8P4G |

^a^ CC _1/2_ = Percentage of correlation between intensities from random half-datasets [1]

^b^ R_sym_ = Σ_hkl_ Σ_i_ |I_i_(hkl) - <I(hkl)>|/ Σ_hkl_ Σ_i_ I_i_ (hkl), where I_i_(hkl) is the observed intensity and <I(hkl)> is the average intensity of multiple observations from symmetry-related reflections [2]

^c^ R_meas_ = Σ_hkl_ [N/(N(hkl) -1)]^1/2^ Σ_i_ |I_i_(hkl) - <I(hkl) >|/Σ_hkl_ Σ_i_ I_i_ (hkl), where N(hkl) is the data multiplicity, I_i_(hkl) is the observed intensity and <I(hkl)> is the average intensity of multiple observations from symmetry-related reflections. It is an indicator of the agreement between symmetry related observations [3]

^d^ R_p.i.m._ = Σ_hkl_ [1/(N(hkl) -1)]^1/2^ Σ_i_ |I_i_(hkl) - <I(hkl) >|/Σ_hkl_ Σ_i_ I_i_ (hkl), where N(hkl) is the data multiplicity, I_i_(hkl) is the observed intensity and <I(hkl)> is the average intensity of multiple observations from symmetry-related reflections. It indicates the precision of the final merged and averaged data set [4]

^e^ R_work_ refers to the actual working data set used in reﬁnement, while R_free_ refers to a cross-validation set that is not directly used in reﬁnement and is therefore free from reﬁnement bias.

**Table S3.** Copper content and molar coefficients at 600 nm for purified wild-type and evolved variants.

| **Enzymes** | **Copper content**  **(mol of Cu/mol of protein)** | **ε_600 nm_**  **(M^-1^ cm^-1^)** |
| --- | --- | --- |
| Wild-type | 3.8 ± 0.2 | 4.2 |
| A209 | 4.0 ± 0.1 | 3.7 |
| G9 | 3.8 ± 0.4 | 4.3 |
| 1B4 | 3.4 ± 0.4 | 3.9 |
| 4B9 | 3.8 ± 0.4 | 4.1 |
| 1B5 | 4.2 ± 0.3 | 3.8 |
| 3F3 | 4.2 ± 0.1 | 4.0 |
| 2G6 | 3.9 ± 0.2 | 3.9 |
| 5F10 | 4.6 ± 0.5 | 3.9 |

**Table S4.** Activity for ABTS measured at 37 °C in buffer pH 4, and sequence analysis of the 13 most active variants after DNA-shuffling between the gene coding for the 1B5 variant and *mcoP* wild-type. Synonymous (S) and non-synonymous (NS) mutations are shown.

| **Variants** | **1B5** | **5E10** | **5C8** | **3F3** | **4D3** | **4G9** | **1G7** | **7G10** | **2G6** | **5F10** | **6B10** | **3E3** | **7F6** | **2G11** |
| --- | --- | --- | --- | --- | --- | --- | --- | --- | --- | --- | --- | --- | --- | --- |
| Vmax (U·mg^-1^) | 0.25 ± 0.05 | 0.85 ± 0.20 | 0.72 ± 0.19 | 0.62 ± 0.20 | 0.61 ± 0.31 | 0.38 ± 0.12 | 0.37 ± 0.06 | 0.30 ± 0.10 | 0.3 ± 0.07 | 0.27 ± 0.05 | 0.27 ± 0.01 | 0.25 ± 0.07 | 0.19 ± 0.04 | 0.18 ± 0.03 |
| V206I | × | × | × | × | × | × | × | × | × | × | × | × | × | × |
| E466G | × |  |  |  |  | × | × | × |  | × |  | × | × | × |
| I259T | × | × | × |  | × |  | × | × | × | × |  | × |  |  |
| M393V | × | × | × | × | × | × | × | × | × |  | × | × | × | × |
| V166I | × | × | × |  | × | × |  | × | × | × | × | × |  | × |
| F361S | × | × | × | × | × | × | × |  |  |  | × | × | × | × |
| P292H | × | × | × | × | × | × | × | × | × | × | × | × | × |  |
| D418N | × |  | × |  | × | × | × | × |  |  | × | × | × | × |
| T38A | × | × |  |  | × | × | × |  |  | × | × |  |  | × |
| N99D | × | × | × |  | × | × | × | × | × | × | × | × | × | × |
| S331P | × | × | × | × | × | × | × | × |  |  | × | × | × | × |
| P390T | × | × | × | × | × | × | × | × | × |  | × | × | × | × |
| (A61) | × | × | × |  | × | × | × | × |  | × | × |  |  | × |
| (P88) | × | × | × |  | × | × | × | × |  | × | × | × |  | × |
| (Y131) | × | × | × |  | × | × |  | × | × | × | × | × |  | × |
| (L150) | × | × | × |  | × | × |  | × | × |  | × | × |  | × |
| (L429) | × |  | × |  | × | × | × | × |  | × | × | × |  | × |
| (V444) | × |  | × |  | × | × | × | × |  |  |  | × |  | × |
| Mutations  (S+NS) | 12+6 | 10+4 | 10+6 | 6+0 | 11+6 | 11+6 | 11+4 | 10+6 | 7+2 | 7+4 | 10+5 | 11+5 | 9+0 | 10+6 |

**Table S5.** Copper content and molar coefficients at 600 nm for wild-type and single variants.

| **Enzymes** | **Copper content**  **(mol of Cu/mol of protein)** | **ε_600 nm_**  **(M^-1^ cm^-1^)** |
| --- | --- | --- |
| Wild-type | 3.8 ± 0.2 | 4.2 |
| E466G | 3.7 ± 0.3 | 5.5 |
| M393V | 3.4 ± 0.1 | 4.4 |
| F361S | 4.3 ± 0.3 | 4.9 |
| P292H | 4.4 ± 0.4 | 4.4 |
| P390T | 4.5 ± 0.5 | 4.9 |

**Table S6.** Apparent steady-state kinetic parameters for ABTS (100 mM sodium acetate, pH 4.5) of purified wild-type and single variants. Reactions were performed at 40 ºC.

| Enzymes | *k*_cat_  (s^-1^) | *K*_m_  (mM) | *k*_cat_/*K*_m_  (s^-1^·M^-1^) |
| --- | --- | --- | --- |
| Wild-type | 1.6 ± 0.02 | 7.1 ± 0.5 | (0.23 ± 0.02) × 10^3^ |
| E466G | 3.2 ± 0.4 | 9 ± 1 | (0.4 ± 0.1) × 10^3^ |
| M393V | 4.2 ± 0.1 | 4.1 ± 0.3 | (1.0 ± 0.1) × 10^3^ |
| F361S | 4.1 ± 0.3 | 14 ± 3 | (0.3 ± 0.1) × 10^3^ |
| P292H | 26 ± 2 | 10 ± 1 | (2.6 ± 0.3) × 10^3^ |
| P390T | 11 ± 1 | 17 ± 1 | (0.7 ± 0.1) × 10^3^ |

**Table S7.** Steady-state kinetic parameters for DMP (100 mM sodium acetate or sodium phosphate at optimal pH) of the purified wild-type and 3F3 variant. Reactions were performed at 40 ºC.

| **Variants** | **pH** | ***k_cat_***  **(s^-1^)** | ***K_m_***  **(mM)** | ***k_cat_*/*K_m_***  **(s^-1^·M^-1^)** |
| --- | --- | --- | --- | --- |
| Wild-type | 8 | 0.019 ± 0.001 | 2.1 ± 0.2 | 9.5 |
| 3F3 | 7.5 | 0.062 ± 0.003 | 0.5 ± 0.1 | 124 |

**Table S8.** Accessible surface areas (ASA, %) of the methionines residues located nearby the T1Cu, in wild-type (PDB 3AW5) and 3F3 variant.

| **Residues** | **ASA (%)** | |
| --- | --- | --- |
|  | **WT** | **3F3** |
| M60 | 29 | 30 |
| M79 | 1 | 1 |
| M145 | 39 | 11 |
| M190 | 37 | 24 |
| M192 | 28 | 7 |
| M232 | 12 | 11 |
| M245 | 0 | 0 |
| M293 | 14 | 14 |
| M353 | 56 | 27 |
| M360 | 25 | 25 |
| M389 | 4 | 6 |
| M400 | 0 | 0 |
| M470 | 0 | 0 |
| M471 | 1 | 0 |

**Table S9.** Dimensions and residues delimiting the tunnels in wild-type (PDB 3AW5) and 3F3 variant. The mutated residues (P/H292 and P/T390) are highlighted in black.

|  | **Wild-type** | **3F3** |
| --- | --- | --- |
| **Residues** | A194, G195, L197, A228, L230, **P292**, H294, E296, G300, M301, E303, P306, E307, M389, **P390**, P392, C460, N462 | M190, E191, A194, G195, L197, A228, **H292,** H294, E296, G298, H299, M301, E303, E307, M389, **T390**, H391, P392, C460, N462 |
| **Length (Å)** | 26.0 | 15.7 |

**Table S10.** Dimensions and residues delimiting the cavities in wild-type (PDB 3AW5) and 3F3 variant.

|  | **Wild-type** | **3F3** | |
| --- | --- | --- | --- |
|  | **Pocket 1** | **Pocket 1** | **Pocket 2** |
| **Residues** | M192, I193, M353, Q354, W355, E464, H465, G468, G469, M470 | M192, I193, M353, Q354, W355, E464, H465, G468, G469 | M190, I193, A194, H294, L295, M297, L350, W355, M389 |
| **Area (Å^2^)** | 210.9 | 219.1 | 187.8 |
| **Volume (Å^3^)** | 133.1 | 118.4 | 105.2 |
| **Depth (Å)** | 10.2 | 8.1 | 10.1 |

**
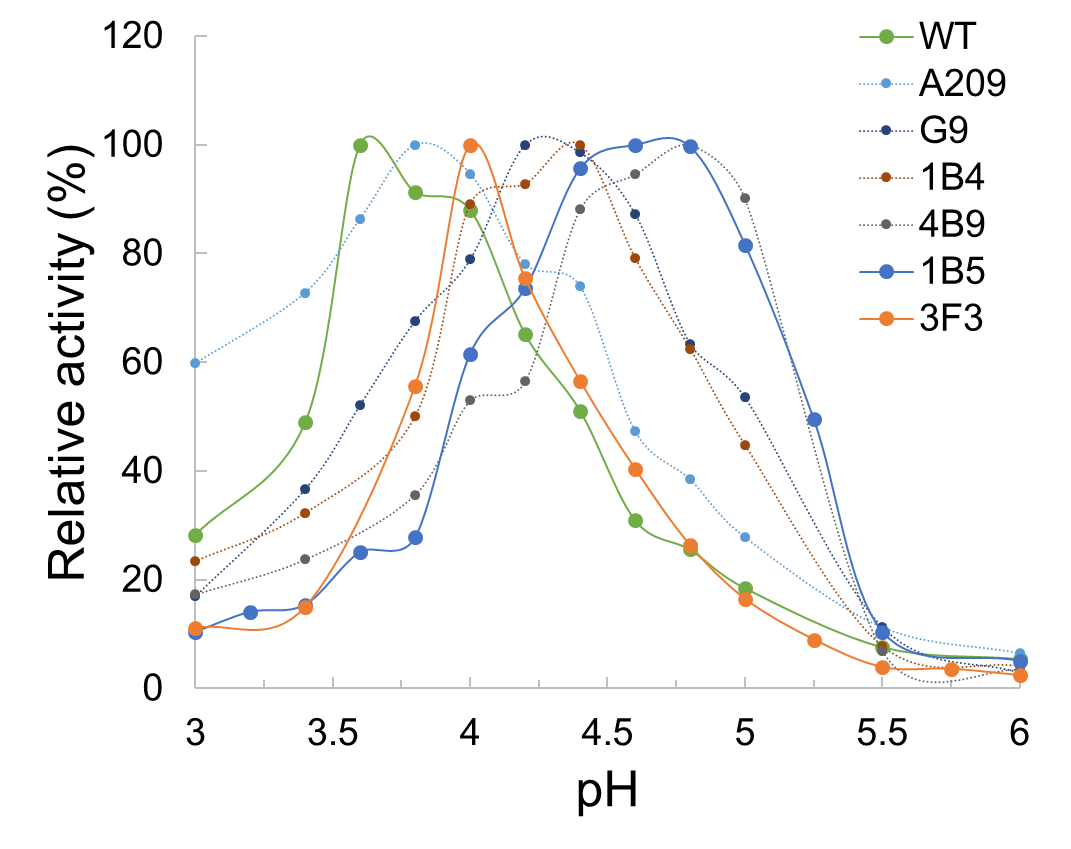
**

**Fig. S1.** pH profile for ABTS of McoP wild type (green) and variants from evolution A209 (light blue), G9 (dark blue), 1B4 (dark red), 4B9 (grey), and 1B5 (blue), 1B5 (blue), and 3F3 (orange).


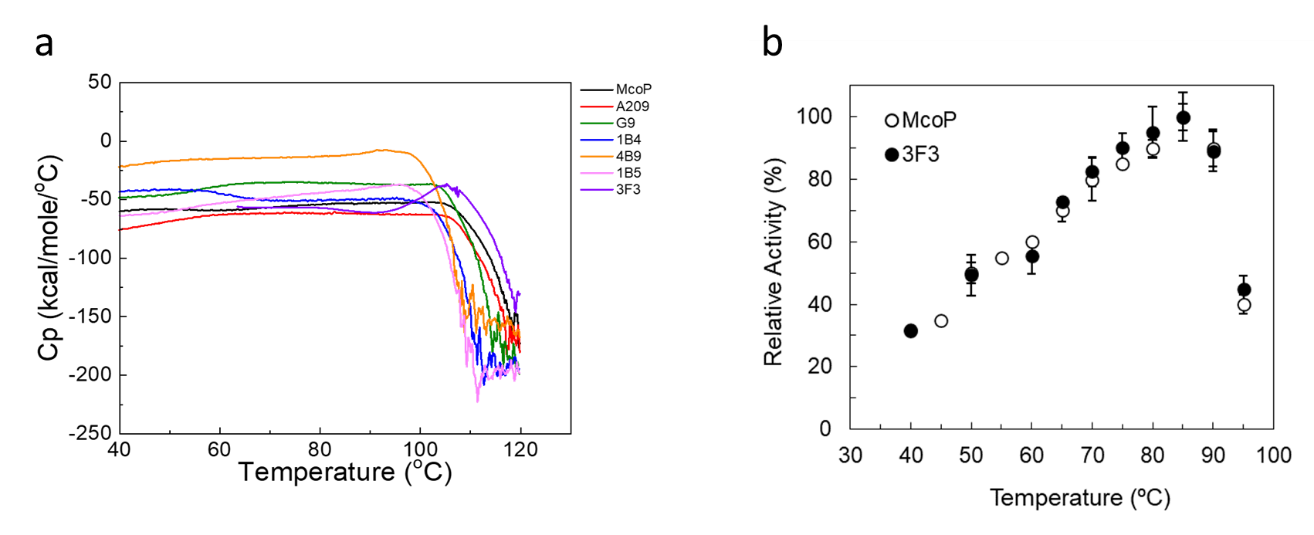


**Fig. S2.** (a) Excess heat capacity of wild-type McoP as its variants obtained from differential scanning calorimetry. Melting temperature (Tm) of wild-type is ~104°C, of variant A209 is ~105 °C, G9 is ~103 °C, 1B4 is ~98 °C, 4B9 is ~96 °C, 1B5 is ~96 °C and 3F3 is ~105 °C. (b) Temperature profile of wild-type McoP (open circle) and 3F3 (closed circle); optimal temperature of both enzymes is 85 °C.


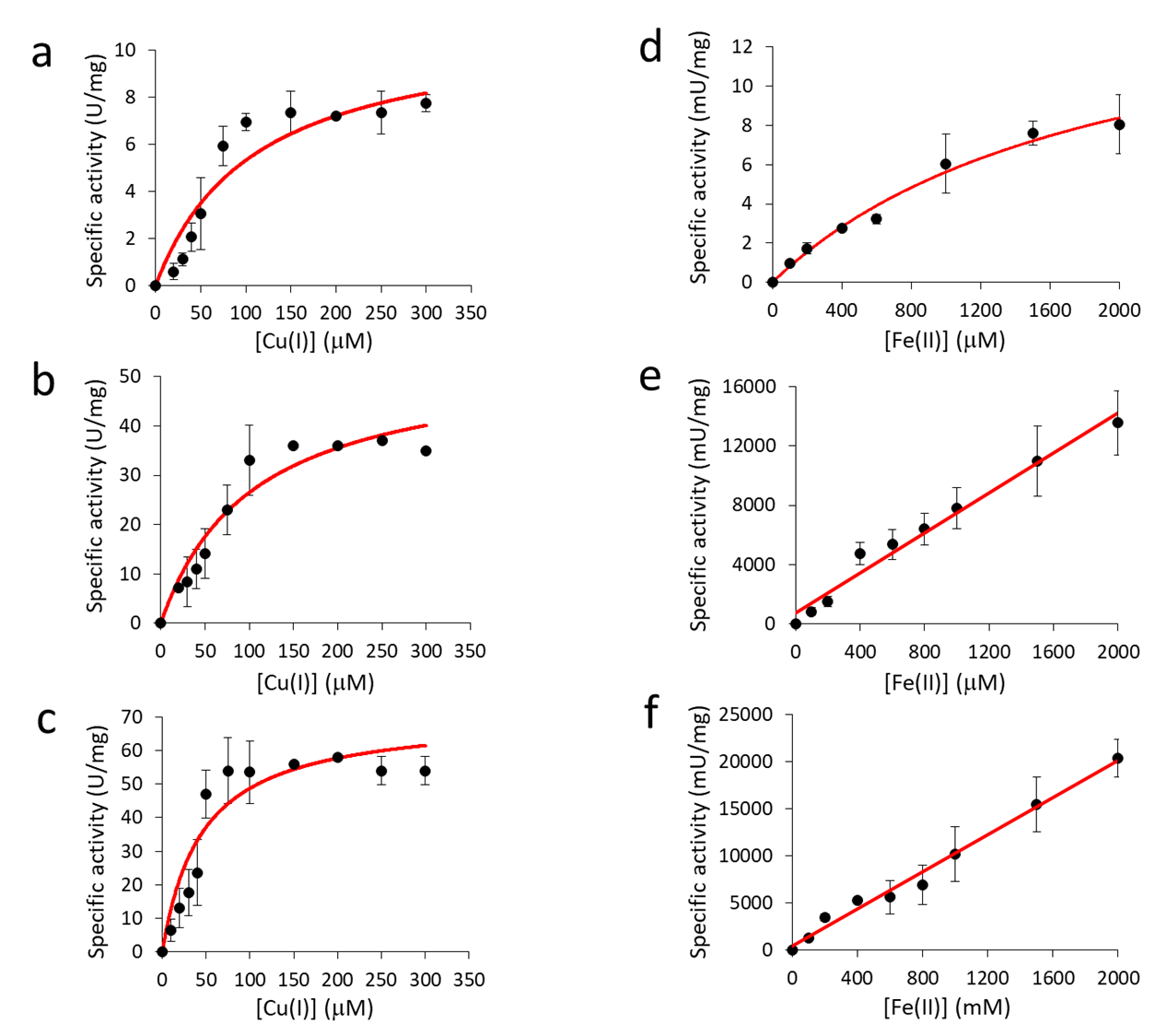


**Fig. S3.** Michaelis-Menten for (a, b, c) Cu(I) oxidation (100 mM sodium acetate buffer, pH 3.5) and (d, e, f) Fe(II) oxidation (100 mM MES buffer, pH 5) of the purified wild-type (a, d), 1B5 (b, e) and 3F3 (c, f) variants. Reactions were performed at 40 ºC.


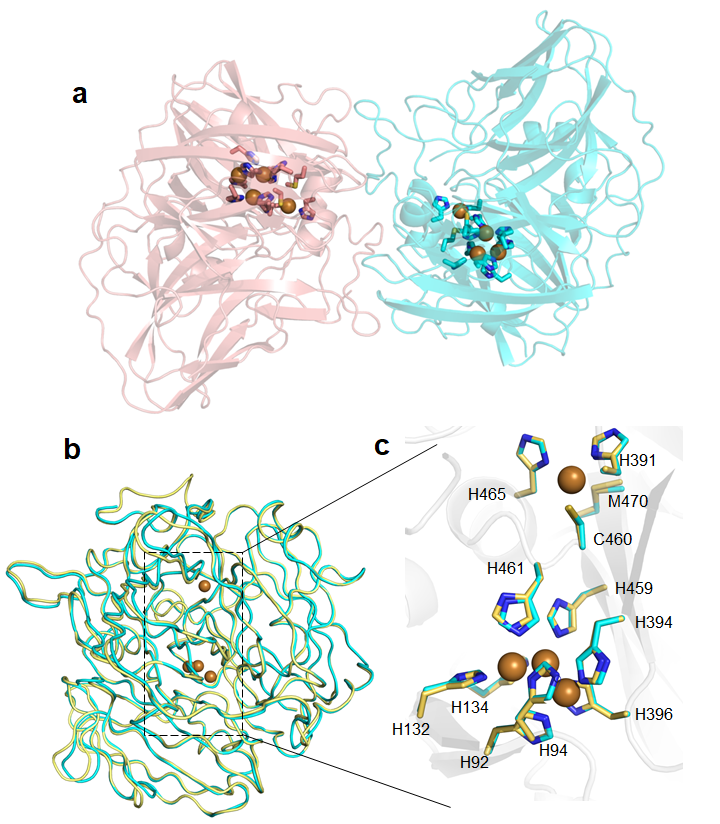


**Fig. S4. Overall structures of 3F3 variant and wild-type. a)** Cartoon representation of chain A (light blue) and chain B (pink) of the 3F3 structure. The T1Cu and TNC ligands are colored in the same color as the corresponding chains. **b)** Structural superimposition of wild-type (orange) and 3F3 (light blue). **c)** Zoomed-view of the T1Cu and TNC sites from wild-type and 3F3, colored as in b). In the wild-type structure, an oxygen atom was modeled in the TNC. In contrast, in 3F3, no electron density was visible in this position, probably due to the lower resolution of the structure (2.59 Å, **Table S2**). The copper ligands are represented as sticks with orange (wild-type) carbon atoms or light blue (3F3), nitrogen, and oxygen atoms colored in blue and red, respectively. The copper atoms are represented as orange spheres.


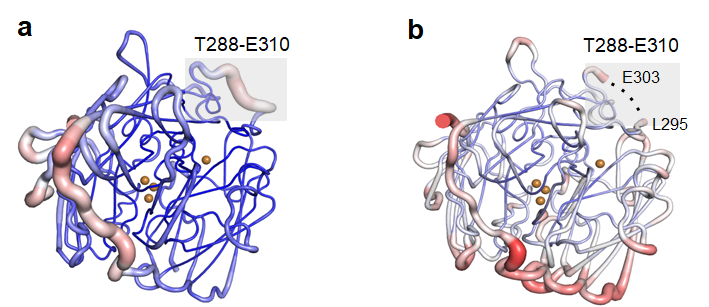


**Fig. S5. Structure B-factor representation of wild-type and 3F3 variant.** Cartoon representation of the main-chain **a)** McoP wild-type, and **b)** 3F3 variant structures with thickness proportional to <a.d.p.> values, color-coded from blue (more rigid) to red (more flexible). The regions with higher flexibility in 3F3 are 154-158, 292-295, 303-305, 319-345, 374-378, 417-420, and 446-453. The copper atoms are shown as orange spheres. The loop T288-E310 is highlighted with a grey box in a) and b). The non-visible region 296-302 in 3F3 is a black dashed line.


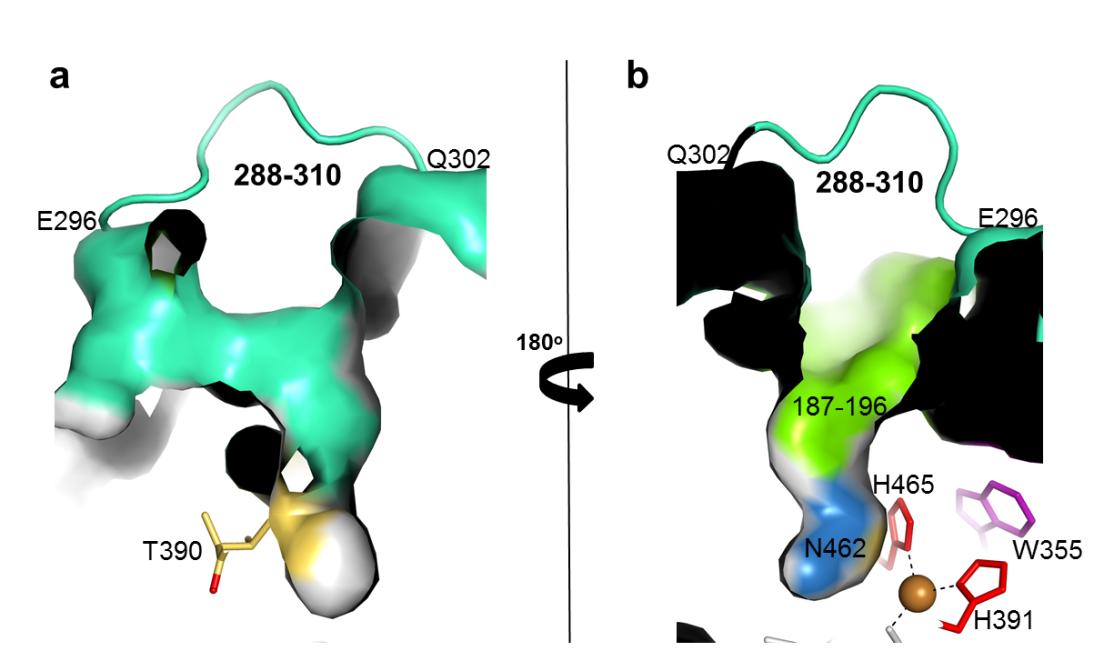


**Fig. S6. Accessible surface area of the 3F3 structure with a highlight of the flexible loop.** A major section of the tunnel is delimited by the residues 288-310 (green cyan), which are part of loop 288-310 a), and 187-196 (green) b). The residues T390 (yellow) and N462 (blue) are located at the bottom of the tunnel. The T1Cu ligands H391 and H465 are shown as red sticks. The W355, located nearby the H465, is shown as a purple stick. The copper atom is represented as an orange sphere.


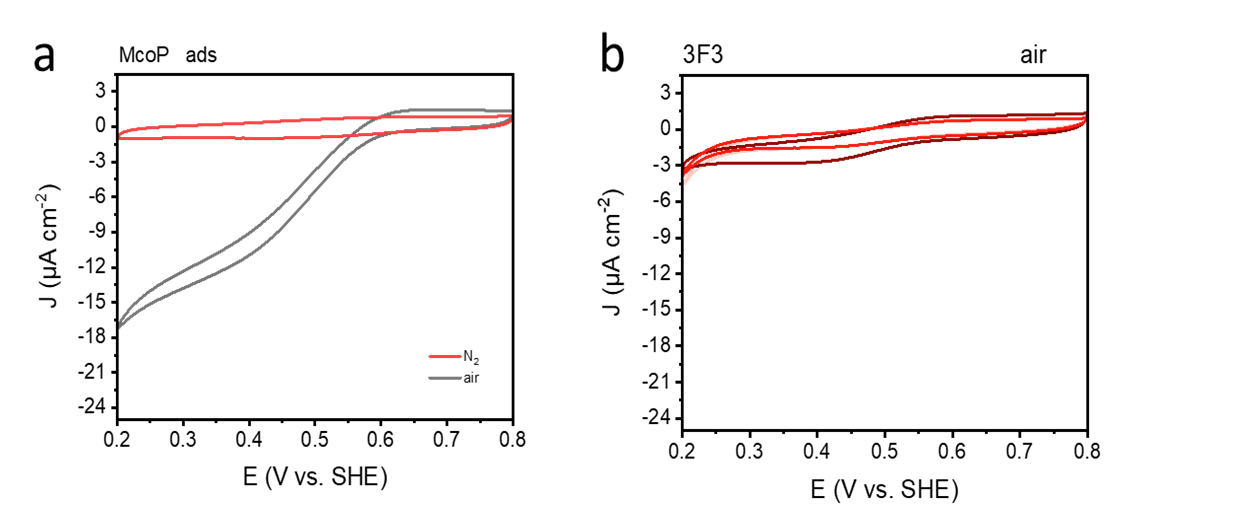


**Fig. S7.** **Electrochemical characterization**. Voltammetric responses under direct electron transfer after immobilizing wild-type (a) and 3F3 (b) in unmodified flat gold electrodes (AuEs). Scan rate: 10 mV·s^−1^. (a) Electrolyte: 0.1 M MES buffer, pH 5.0. Red line: N_2_ atmosphere, gray line: ambient conditions; (b) Electrolyte: phosphate-citrate buffer, pH 5.0; ambient conditions. The different cycles in (b) correspond to two independently prepared electrodes for the 3F3 variant.


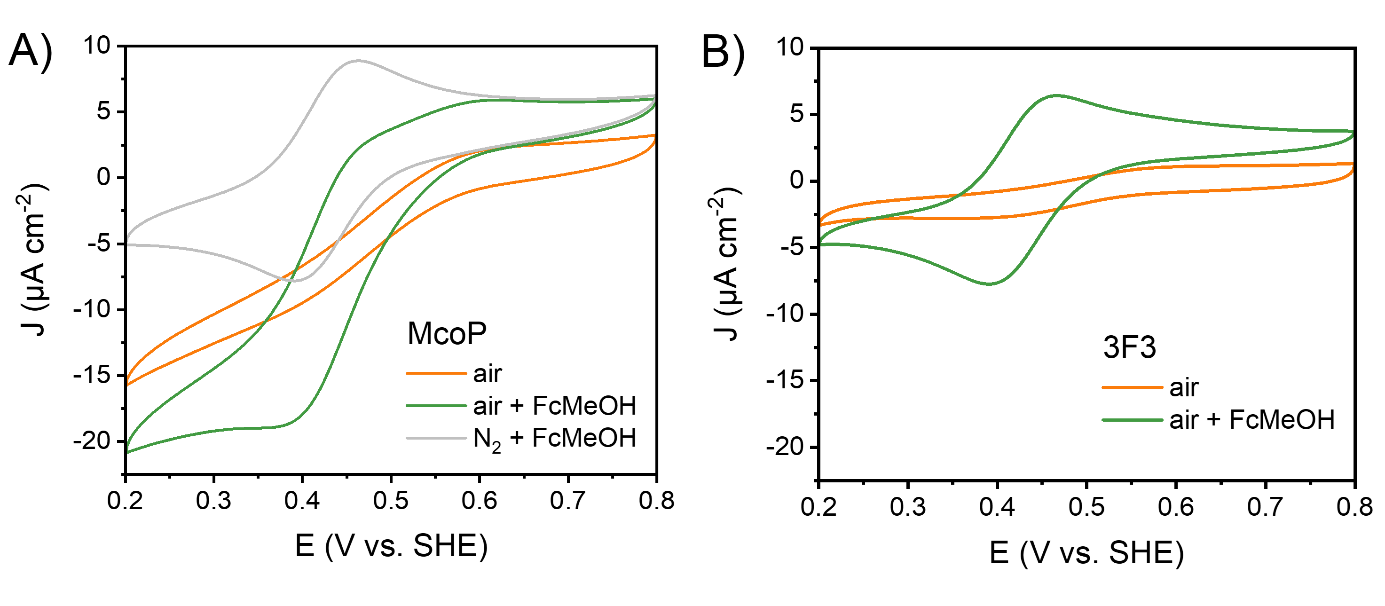


**Fig. S8.** Electrochemical characterization of electrodes modified with McoP WT (A) or the variant 3F3 (B) in the absence or presence of the redox mediator ferrocenemethanol (FcMeOH) under air atmosphere. FcMeOH presents a midpoint potential of about 0.44 V vs. SHE, as indicated by the response obtained under an N2 atmosphere (gray curve in panel A). Scan rate: 10 mV s^−1^. Electrolyte: phosphate-citrate buffer, pH 5.0; [FcMeOH] = 100 µM.


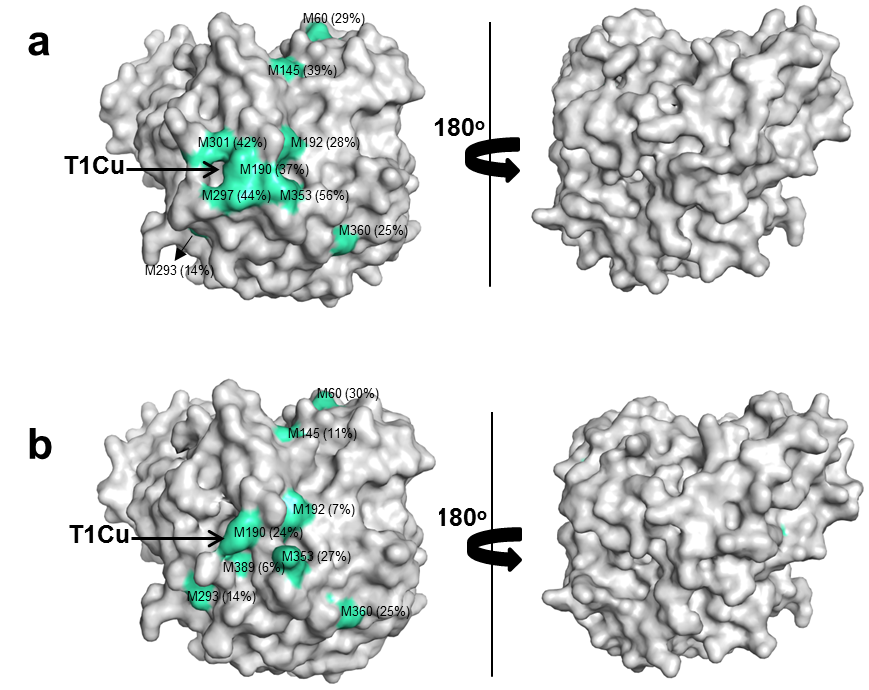


**Fig. S9. Accessible surface area showing solvent-exposed methionines in wild-type and 3F3 variant.** The solvent-exposed methionines are colored in green cyan in wild-type a) and 3F3 variant b). The methionine residues are labeled, and their accessible surface areas are shown in parenthesis. The location of the T1Cu is highlighted in both a) and b). A 180 degrees rotation in the Y axis revealed no methionines exposed on the opposite side of the T1Cu.


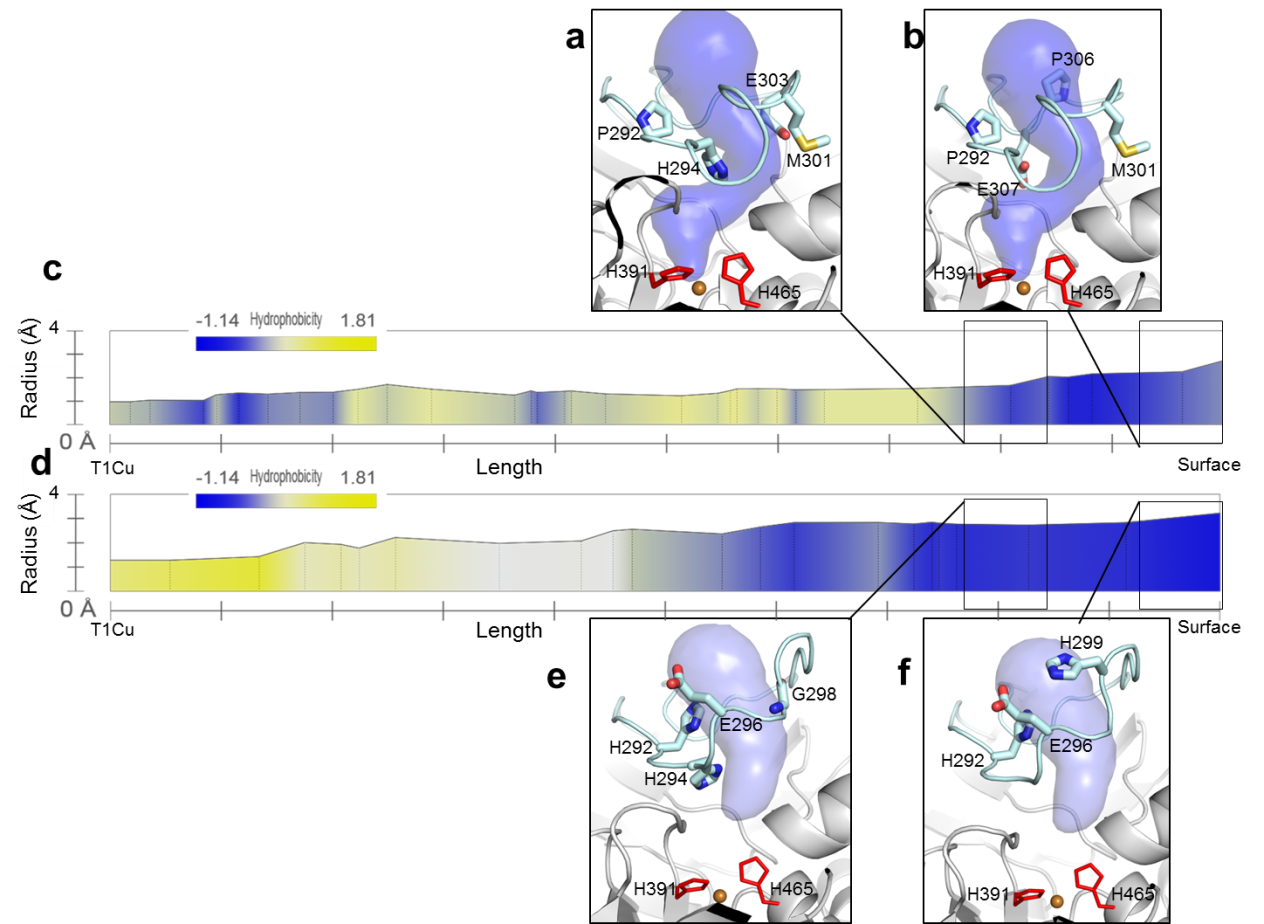


**Fig. S10. Properties of the tunnel that connects the T1Cu with the solvent.** The carbon atoms of the residues that delimit the entrance area of the tunnel in wild-type a) and b) and in 3F3 variants e) and f) are colored in cyan. The hydrophobicity characteristics across the tunnel in wild-type c) and 3F3 d) are ranked from blue (more polar) to yellow (more apolar). The tunnel's radius (Å) and length (Å) are also shown. The left side of the tunnel corresponds to the bottom part, located in the T1Cu, and the right side corresponds to the top part on the outside surface. The oxygen, nitrogen, and sulfur atoms are shown as sticks colored red, blue, and yellow, respectively. The T1Cu ligands (H391 and H465) are shown as red sticks. The copper atom is shown as an orange sphere.

**
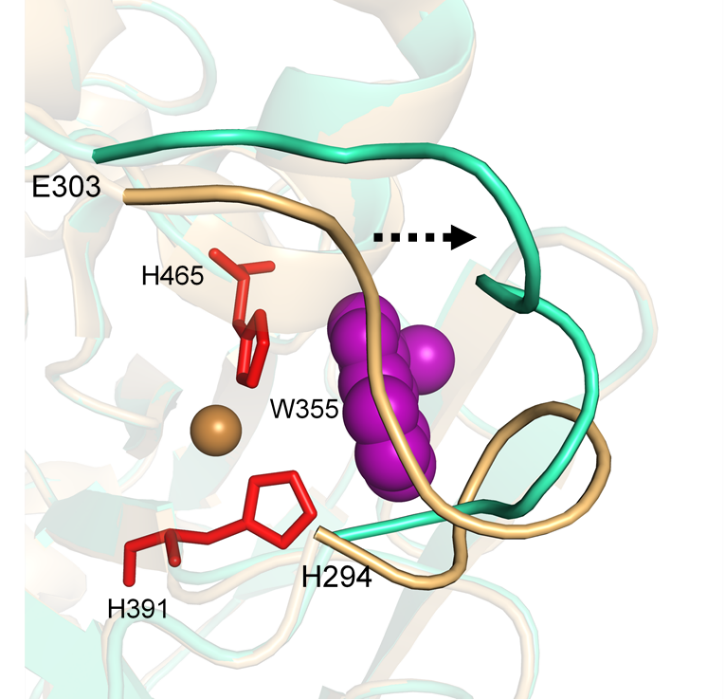
**

**Fig. S11. Structural displacement of loop 288-310 in 3F3.** The loop in the wild-type and 3F3 is colored in beige and pale green, respectively. The black arrow indicates the movement of the loop that in 3F3 allows the creation of pocket 2, exposing the W355 side-chain (purple spheres) to the solvent. The T1Cu ligands (H391 and H465) are shown as red sticks. The copper atom is shown as an orange sphere.

Creation of a new pocket (P2) in 3F3

**
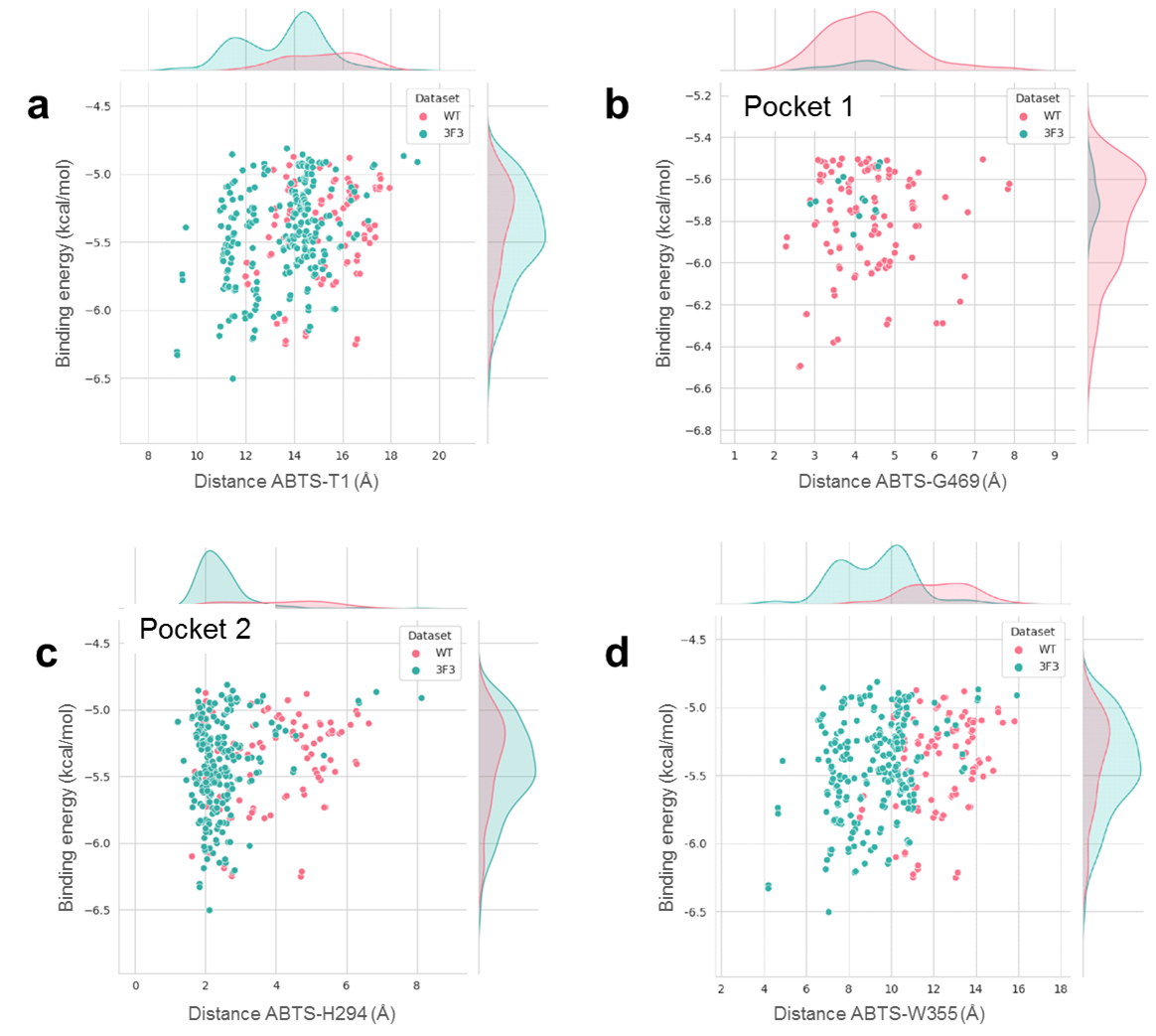
**

**Fig. S12. ABTS docking simulations in wild-type and 3F3.** Binding energy (kcal/mol) *vs.* distances (Å) for ABTS-T1Cu a), ABTS-G469 b), ABTS-H294 c) and ABTS-W355 d). Each point represents a simulated conformation of the enzyme-substrate complex showing the docking poses in wild-type (pink) and 3F3 (blue).

**REFERENCES**

1. Karplus PA & Diederichs K (2012) Linking Crystallographic Model and Data Quality. Science 336:1030-1033.

2. Arndt UW, Crowther RA & Mallett JFW (1968) A Computer-Linked Cathode-Ray Tube Microdensitometer for X-Ray Crystallography. J Phys E Sci Instrum 1:510-516.

3. Diederichs K & Karplus PA (1997) Improved R-factors for diffraction data analysis in macromolecular crystallography. Nat Struct Biol 4:269-275.

4. Weiss MS (2001) Global indicators of X-ray data quality. J Appl Crystallogr 34:130-135.
